# A Comprehensive Study of CO_2_ Absorption and Desorption by Choline-Chloride/Levulinic-Acid-Based Deep Eutectic Solvents

**DOI:** 10.3390/molecules26185595

**Published:** 2021-09-15

**Authors:** Mohaned Aboshatta, Vitor Magueijo

**Affiliations:** Chemical & Process Engineering Department, University of Strathclyde, Glasgow G1 1XJ, UK; vitor.magueijo@strath.ac.uk

**Keywords:** deep eutectic solvents, choline chloride, levulinic acid, CO_2_ absorption, DES regeneration, carbon capture, green absorbents

## Abstract

Amine absorption (or amine scrubbing) is currently the most established method for CO_2_ capture; however, it has environmental shortcomings and is energy-intensive. Deep eutectic solvents (DESs) are an interesting alternative to conventional amines. Due to their biodegradability, lower toxicity and lower prices, DESs are considered to be “more benign” absorbents for CO_2_ capture than ionic liquids. In this work, the CO_2_ absorption capacity of choline-chloride/levulinic-acid-based (ChCl:LvAc) DESs was measured at different temperatures, pressures and stirring speeds using a vapour–liquid equilibrium rig. DES regeneration was performed using a heat treatment method. The DES compositions studied had ChCl:LvAc molar ratios of 1:2 and 1:3 and water contents of 0, 2.5 and 5 mol%. The experimental results showed that the CO_2_ absorption capacity of the ChCl:LvAc DESs is strongly affected by the operating pressure and stirring speed, moderately affected by the temperature and minimally affected by the hydrogen bond acceptor (HBA):hydrogen bond donator (HBD) molar ratio as well as water content. Thermodynamic properties for CO_2_ absorption were calculated from the experimental data. The regeneration of the DESs was performed at different temperatures, with the optimal regeneration temperature estimated to be 80 °C. The DESs exhibited good recyclability and moderate CO_2_/N_2_ selectivity.

## 1. Introduction

Renewable electricity production methods are still not an alternative to the burning of fossil fuels, irrespective of their progressive advances. Anthropogenic emissions of greenhouse gases (GHGs) are anticipated to continue increasing in the coming decades, with anthropic CO_2_ expected to remain one of the primary reasons behind climate change.

Over the last six decades, the concentration of CO_2_ in the atmosphere has been increasing dramatically, reaching a value of approximately 418 ppm in June 2021 [1]. Amine absorption is currently the most applied and established method for CO_2_ capture [2]. The method utilises conventional amines (typically monoethanolamine, MEA), which have environmental shortcomings and consume a lot of energy to regenerate the solvent [3]. Deep eutectic solvents (DESs) are among the favourable alternatives to substitute conventional amines for CO_2_ capture and have some advantages when compared to ionic liquids. DESs are greener and more benign solvents for CO_2_ capture than ionic liquids due to their biodegradability, lower toxicity and lower prices [4,5,6].

DESs are typically hygroscopic systems and therefore always contain a certain amount of water. The effect of the water content on CO_2_ absorption by a DES (CO_2_ solubility) depends on the specific composition of the DES, with researchers reporting different trends for different systems. Several researchers [7,8,9] have studied the CO_2_ absorption in choline-chloride-based DESs with urea (reline), with the review by Rima et al. [10] showing reline as a promising sorbent for CO_2_ capture when compared to ILs. However, CO_2_ solubility in reline drops significantly with increasing water content as water acts as an anti-solvent in this case [11,12]. On the other hand, CO_2_ solubility increases with water content in other DES systems. For example, this behaviour is observed in L-arginine:glycerol mixtures with molar ratios ranging between 1:4 and 1:8, as reported by Ren et al. [13]. One potential explanation for this phenomenon is that small increments in water content lead to a significant reduction in the viscosity of the DESs, which enhances CO_2_ solubility [13].

Choline chloride (ChCl):levulinic acid (LvAc) DESs are biodegradable, non-toxic and cheap sorbents, as reported by Magugeri et al. [14] CO_2_ absorption into ChCl:LvAc DESs has been studied at a molar ratio of 1:2, at a temperature of 50 °C and at pressures up to 30 bar by Ullah et al. [15], while Lu et al. increased the molar ratio of the hydrogen bond donor (levulinic acid) up to 1:5 [16]. However, the effect of water on CO_2_ solubility in ChCl:LvAc is still unknown; because both the HBD and the HBA are hygroscopic [17], studying the effect of water on CO_2_ solubility is crucial for practical reasons when dealing with wet flue gas as well. Besides, CO_2_ absorption thermodynamics in ChCl:LvAc DESs and sorbent/DES regeneration are still not reported in the literature, as concluded by the authors in a recent review of the literature (unpublished work).

Many studies in the literature have reported on the effect of stirring speed on CO_2_ absorption by ethanolamine aqueous solutions [18,19]. For example, Pashaei et al. [20] reported a dramatic increase in the CO_2_ absorption capacity of aqueous solutions of diethanolamine (DEA) by increasing the stirring speed of their apparatus from 0 to 600 rpm. On the other hand, no such attention has been given to the effect of stirring speed on CO_2_ absorption by deep eutectic solvents (DESs). For example, although Ullah et al. [15] and Lu et al. [16] have reported the CO_2_ absorption of some choline chloride:levulinic acid DESs at different temperatures and pressures, the effect of the stirring speed on the CO_2_ absorption capacity was not taken into consideration. For a better assessment of the viability of any sorbent for CO_2_ capture purposes, the absorption isotherms should be studied under dynamic conditions similar to ones applied in industrial plants (i.e., in the absorption columns), rather than studying the absorption capacity of sorbents under stagnant conditions.

Few researchers in the literature have reported on the recyclability of the DESs. For example, Ren et al. [13] studied the regeneration process of L-arginine:glycerol DESs with a molar ratio of 1:6 by bubbling nitrogen gas at 100 °C for 80 min to release the CO_2_. The recyclability of choline chloride:levulinic acid DESs is still unknown; therefore, one of the main objectives of this work is to investigate the recyclability of ChCl:LvAc DESs at constant conditions of temperature, pressure and stirring speed.

Most researchers in the literature have reported the solubility of acidic gases [21] such as hydrogen sulfide [22], nitrogen dioxide [23], methane [22] and CO_2_ gas mixtures in the DESs. However, for most DESs the selectivity of DESs towards CO_2_ in a mixture of CO_2_/N_2_ gases is not commonly tested, including the CO_2_/N_2_ selectivity of choline-chloride:levulinic-acid-based DESs.

The major objective of this study is to investigate CO_2_ absorption and desorption at different operating conditions. Experimental parameters such as the HBA:HBD molar ratio, pressure, temperature, stirring speed and water content were selected as input factors and their effect was investigated. The experimental results were also used to calculate the thermodynamic properties of CO_2_ absorption in ChCl:LvAc DESs. Additionally, this work aims to study the recyclability of ChCl:LvAc DESs and their selectivity towards CO_2_ at specific conditions of temperature, pressure and stirring speed.

## 2. Materials and Methods

### 2.1. Preparation of the DESs

Choline chloride (>99% *w/w*) and levulinic acid (>98% *w/w*) were supplied by Fisher Scientific, Loughborough, UK. These chemicals were used without any further purification to prepare DESs. In this work, the DESs were prepared by the heating method that has been commonly used in the literature ([3,7,13,24,25,26,27,28]). The two components (the HBA and the HBD) were mixed at different molar ratios of 1:2 and 1:3 with different water contents, –0, 2.5 and 5%, respectively. The mixture was then heated at 25 °C under constant stirring until a homogeneous system was formed. The materials were weighed using a Mettler Toledo AM100 electronic balance with an accuracy of 1 × 10^−4^ g. The water content of the prepared DESs was measured using a Karl Fisher moisture titrator from Kyoto Electronics Ltd., model MKH-700.

In this work, the composition of a DES is represented by a label of the type “(*HBA:HBD:water molar percentage*)”. As an example, a DES with label “(1:2:5)” is a DES with a composition defined by an HBA/HBD molar ratio of 1:2 and 5 mol% of water. This nomenclature avoids the unnecessary lengthy reference to the water content each time a specific DES composition is mentioned.

### 2.2. CO_2_ Absorption Measurements

**Figure 1:**
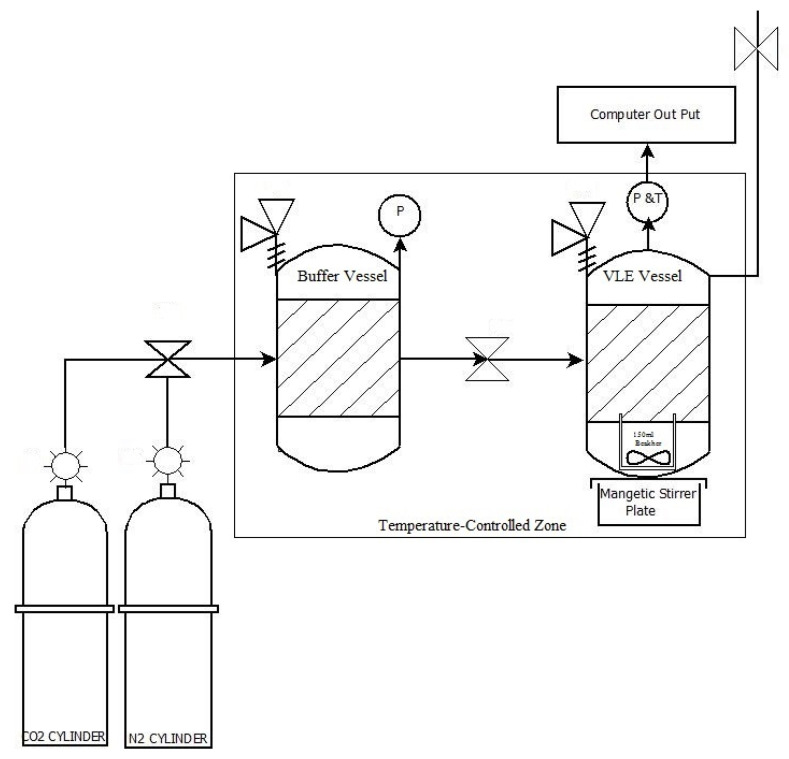
Schematic diagram of the VLE absorption rig to measure CO_2_ absorption by DES systems. Adopted with modification from [30].

A vapour–liquid equilibrium (VLE) absorption rig was used to conduct the CO_2_ absorption experiments—see Figure 1 below. The VLE rig consisted of a buffer vessel and a VLE measurement vessel (Millipore pressure vessels, model XX6700P120). Both vessels were fitted with pressure gauges and valves to monitor and control the pressure. An incubation chamber was used to control the temperature of the system with a precision of ±0.5 °C. The pressure and temperature inside the absorption vessel was monitored by a GS4200-USB digital pressure–temperature transducer and software purchased from ESI Technology Ltd., Wrexham, UK.

The CO_2_ absorption capacity of different ChCl:LvAc DESs was measured at different pressures, temperatures and stirring speeds. In the absorption measurement runs, a known mass of DES (40 g) was loaded into the VLE measurement vessel. After thermal equilibrium was reached—normally within 2 h—the pure CO_2_ (or pure N_2_ and CO_2_/N_2_ mixtures in the case of the selectivity tests) was quickly transferred from the buffer vessel into the VLE measurement vessel until the desired pressure level was reached. The system was maintained under test conditions (temperature and stirring rate) until equilibrium was reached.

To ensure the validity of results, baseline and calibration runs were carried out before conducting any absorption measurements. The baseline runs were “leakage detection runs” and consisted of charging the rig with CO_2_ in the absence of DES at a constant temperature, and in monitoring the pressure drop in the system until equilibrium was reached. Baseline runs were necessary to ensure that the pressure drop in the system during the measurement runs was only due to gas absorption by the DESs and not due to residual leakages. The calibration runs consisted in charging the rig with an aqueous solution of MEA 30 wt% (instead of a DES) and by running CO_2_ absorption experiments at 25 °C and at pressures up to 6 bar. The results of the baseline and calibration runs are available in Figures S1–S6 in the supplementary information.

Generally, DESs have low vapour pressures [29]; therefore, it was assumed that the total pressure in the system is equal to the pressure of the pure gas or gas mixture charged into the rig. The CO_2_ absorption capacity by the ChCl:LvAc DES was calculated based on the absolute pressure drop due to absorption only, as per Equations (1) and (2) below, and then expressed in terms of moles of CO_2_ per kg of DES (mol·kg^−1^). The pressure drop due to CO_2_ absorption only, ΔP, is given by:

(1) $$∆P=\left(P_{{\mathrm{CO}}_{2}}^{\circ }-P_{{\mathrm{CO}}_{2}}^{\mathrm{equil}}\right)-\left(P_{\mathrm{baseline}}^{\circ }-P_{\mathrm{baseline}}^{\mathrm{equil}}\right)$$

The first term of Equation (1) represents the pressure drop in the system during the CO_2_ absorption runs (measurement runs), whereas the second term accounts for the pressure drop in the system due to leakages (baseline runs). In Equation (1), $P_{{\mathrm{CO}}_{2}}^{\circ }$ is the CO_2_ pressure at the start of the absorption/measurement run, $P_{{\mathrm{CO}}_{2}}^{\mathrm{equil}}$ is the CO_2_ pressure when the system reaches equilibrium during the measurement run, $P_{\mathrm{baseline}}^{\circ }$ is the pressure at the start of the baseline run and $P_{\mathrm{baseline}}^{\mathrm{equil}}$ is the pressure when the system reaches equilibrium during the baseline run. The experimental results (raw data) are available in the supplementary information.

The values of pressure drop were then used to calculate the number of moles of CO_2_ absorbed by 40 g of the DES:

(2) $$n=\frac{V\;*∆P}{R*T}$$

where n is the number of CO_2_ moles absorbed by the DES, V is the volume of the measurement vessel in m^3^, ∆P is the absolute pressure drop due to absorption calculated using Equation (1) (in Pa), T is the absorption temperature in Kelvin and R is the universal gas constant in J mol^−1^ K^−1^.

### 2.3. Thermodynamic Analysis of CO_2_ Absorption

The solubility of CO_2_ in the DESs can be expressed in terms of Henry’s law constant. The smaller the value of the Henry’s law constant, the higher the solubility in the liquid [7,31,32]. The Henry’s law constant based on mass fractions, H_x_, was calculated using Equation (S4) in the supplementary information. Additionally, the enthalpy (∆H), Gibbs free energy (∆G) and entropy (∆S) of absorption were calculated using the Van ’t Hoff Equations ((S5)–(S7) in the supplementary information).

### 2.4. Sorbent Regeneration Measurements

Sorbent regeneration starts once the rig is depressurised, with the spontaneous release of part of the absorbed gas. To release the residual CO_2_ still absorbed in the DES samples after depressurisation (CO_2_ not spontaneously released), a heat treatment was used. CO_2_ desorption tests were performed in an oven by heating 10 g aliquots of the DES samples with residual CO_2_ at temperatures of 60 °C, 80 °C and 100 °C for 60 min. The weight loss method was used, with the weight loss of the aliquots measured by a Mettler Toledo AM100 electronic balance with an accuracy of 1 × 10^−4^ g.

### 2.5. FTIR Spectra Measurements

To determine the CO_2_ released after thermal treatment at each desorption temperature, FTIR spectra of the DES samples were measured before CO_2_ absorption, after CO_2_ absorption and after thermal treatment. The FTIR spectra were measured using an ABB MB 3000 FTIR spectrophotometer (Clairet Scientific Ltd., Northampton, UK). The device was calibrated with air before measuring the transmittance of the samples. The CO_2_ released was determined using the Beer–Lambert law [33] as per Equation (3) below:

(3) A= ε L C

where A is the absorbance of samples before and after CO_2_ absorption and after desorption at different temperatures, ε is the molar absorptivity of CO_2_ in (L·mol^−1^·cm^−1^), L is the path length of the sample in cm and C is the concentration of CO_2_ in the sample in mol·L^−1^.

### 2.6. Recyclability of the DESs

Two replicate samples of ChCl:LvAc (1:2:0) were selected for this purpose. The DES samples were subjected to 5 sequential absorption/desorption cycles under the following experimental conditions: constant temperature of 25 °C; CO_2_ pressures up to 300 kPa during the absorption step; 250 rpm stirring speed during the absorption step; and desorption (heat treatment) temperature of 80 °C.

All samples were cooled down to room temperature before starting the next absorption/desorption cycle. Weight gain and weight loss measurements were taken before and after each CO_2_ absorption and desorption step, respectively.

### 2.7. Selectivity of the DESs towards CO_2_

Samples of ChCl:LvAc DES with composition 1:3:2.5 were used in the CO_2_ selectivity tests. The tests were performed using N_2_/CO_2_ gases mixtures with different molar ratios of CO_2_ (100%, 50%, 15% and 0%, respectively). All tests were carried out under constant temperature of 25 °C, pressures up to 300 kPa and stirring speed 250 rpm. The total pressure inside the buffer vessel is equal to the summation of the partial pressures of the two gases. The partial pressures of CO_2_ and N_2_ before and at the start of the absorption measurements are given in Table 1.

The absorption of the pure CO_2_ is taken as the baseline in this case. The selectivity of absorbing CO_2_ over N_2_ of the DESs is defined as ($S_{{\mathrm{CO}}_{2}/N_{2}}$) as per Equations (4) and (5) below:

Selectivity based on the absorption of pure gases in the DES:

(4) $$S_{{\mathrm{CO}}_{2}/N_{2}}=\frac{x_{{\mathrm{CO}}_{2}}}{x_{N_{2}}}$$

Selectivity based on the absorption of mixtures of gases in the DES:

(5) $$S_{{\mathrm{CO}}_{2}/N_{2}}=\frac{x_{{\mathrm{CO}}_{2}}}{x_{N_{2}}}\times \frac{y_{N_{2}}}{y_{{\mathrm{CO}}_{2}}}$$

where *x* is the mole fraction of CO_2_ or N_2_ absorbed by the DESs, and *y* is the mole fraction of CO_2_ or N_2_ in the gas phase.

## 3. Results and Discussions

### 3.1. CO_2_ Absorption

**Figure 2:**
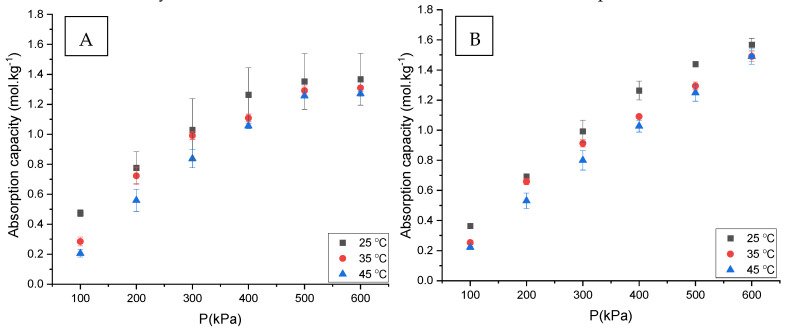

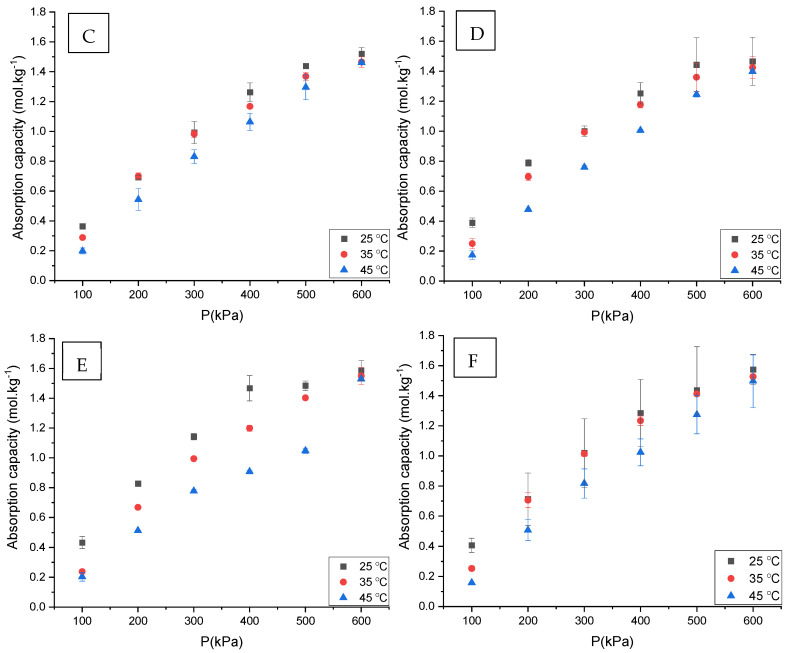
CO_2_ absorption capacity of ChCl:LvAc DESs with different compositions at temperatures ranging from 25 °C to 45 °C and at a constant stirring speed of 250 rpm: (**A**)—1:2:0; (**B**)—1:2:2.5; (**C**)—1:2:5; (**D**)—1:3:0; (**E**)—1:3:2.5; and (**F**)—1:3:5.

In the first set of experiments, the CO_2_ absorption capacity of different ChCl:LvAc DESs was measured at different pressures and temperatures, with the DESs under a constant stirring speed of 250 rpm. The results obtained are presented in Figure 2, which shows quite similar values of absorption capacity and trends for all DES compositions. A visual inspection of Figure 2 seems to indicate that, overall, the HBA:HBD molar ratio and the water content both have a small effect on the CO_2_ absorption capacity in ChCl:LvAc DESs. The only obvious trend observed concerns the effect of temperature, with the CO_2_ absorption capacity decreasing with increasing temperature for all compositions. The temperature trend observed for the ChCl:LvAc DESs agrees with the results reported in the literature for other DESs [7,30,31].

**Figure 3:**
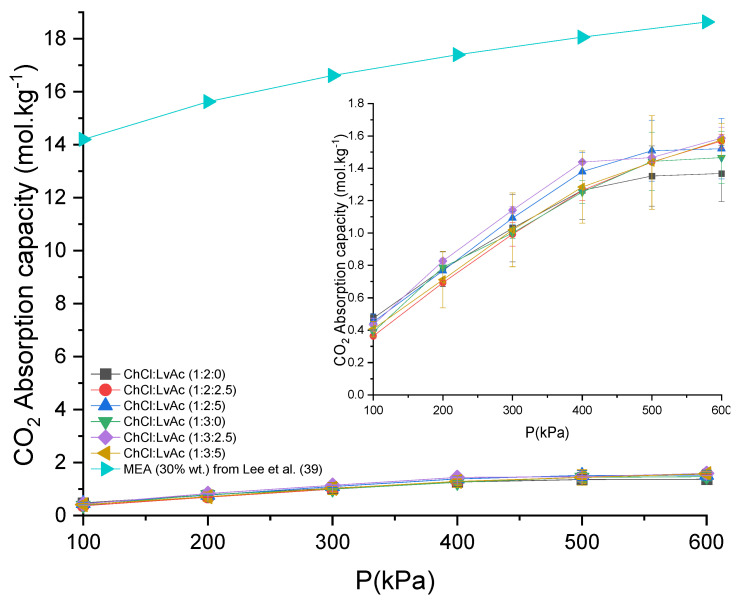
The CO_2_ absorption capacity of ChCl:LvAc vs. MEA 30% wt. at 25 °C.

The CO_2_ absorption capacities measured for the ChCl:LvAc DESs are clearly lower than the CO_2_ absorption capacity reported in the literature for aqueous solutions of MEA at 30 wt% [34], as shown in Figure 3. This might be due to the fact that the CO_2_ absorption mechanism is different in both cases: CO_2_ absorption in MEA solutions is a combination of chemisorption and physisorption [35,36], whereas the CO_2_ absorption in the DESs studied in this work is limited to physisorption only [37].

The values of CO_2_ absorption capacity reported in this work are higher than the values reported in the literature by Ullah et al. [15] and Lu et al. [16] for ChCl:LvAc DESs at pressures between 1 and 6 bar. The justification for the higher values presented in this work seems to be related with the use of “flow”/dynamic conditions. While the results reported by Ullah et al. [15] and Lu et al. [16] seem to have been measured under stagnant conditions, the results presented in Figure 2 were all obtained under strong stirring (250 rpm). To support this hypothesis, the effect of stirring speed on the CO_2_ absorption capacity of ChCl:LvAc DESs was studied and the results are presented in Section 3.3.

### 3.2. Thermodynamic Analysis of CO_2_ Absorption

According to the literature, the absorption of CO_2_ in DESs made of choline chloride and carboxylic acids is only due to physisorption [37]. The thermodynamic properties of CO_2_ absorption in ChCl:LvAc DESs were estimated at 1 bar. Henry’s law constant (Hx), the change in enthalpy (∆H), the change in entropy (∆S) and the change in Gibbs free energy (∆G) were calculated and are presented in Table 2.

**Figure 4:**
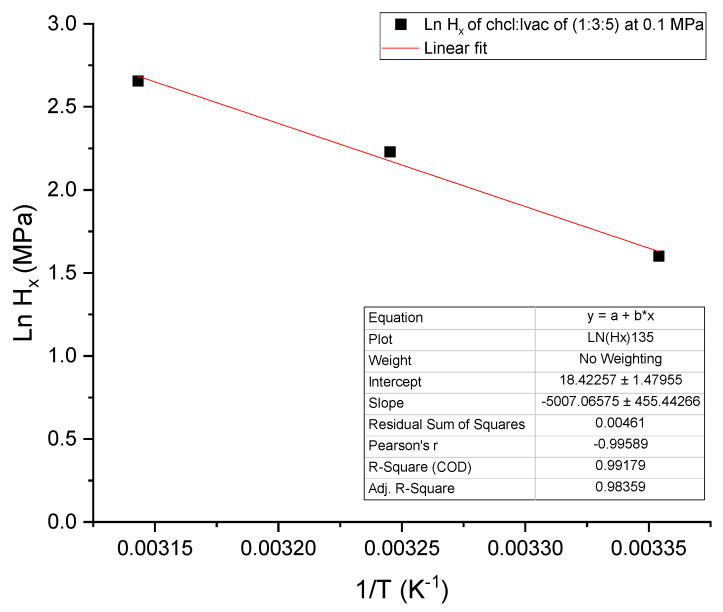
Arrhenius plot of Henry’s law constant for the absorption of CO_2_ in ChCl:LvAc (1:3:5).

As expected based on the experimental results presented in Section 3.1, the values of Hx tabulated in Table 2 are very consistent and similar for all DES compositions. The values of Hx increase with increasing temperature for all ChCl:LvAc compositions and the data are well fitted by a ln(Hx) versus 1/T correlation as exemplified by the Arrhenius plot in Figure 4.

CO_2_ absorption in ChCl:LvAc DESs is exothermic (∆H < 0); therefore, increasing the temperature decreases CO_2_ uptake as reported in the literature by other researchers [16,38]. The negative change of entropy (∆S < 0) for CO_2_ absorption in ChCl:LvAc DESs indicates a more ordered system after absorption takes place [39]. Additionally, the ∆G values for the CO_2_ absorption in ChCl:LvAc DESs were found to be positive, signifying that the absorption process is not thermodynamically spontaneous. The results obtained are aligned with results reported in the literature for other DESs. For example, Mirza et al. [30] analysed the thermodynamics of CO_2_ absorption in reline, ethaline and malinine, and showed that the absorption process was also exothermic and nonspontaneous.

### 3.3. The Effect of Stirring Speed on the CO_2_ Absorption Capacity of the DES

**Figure 5:**
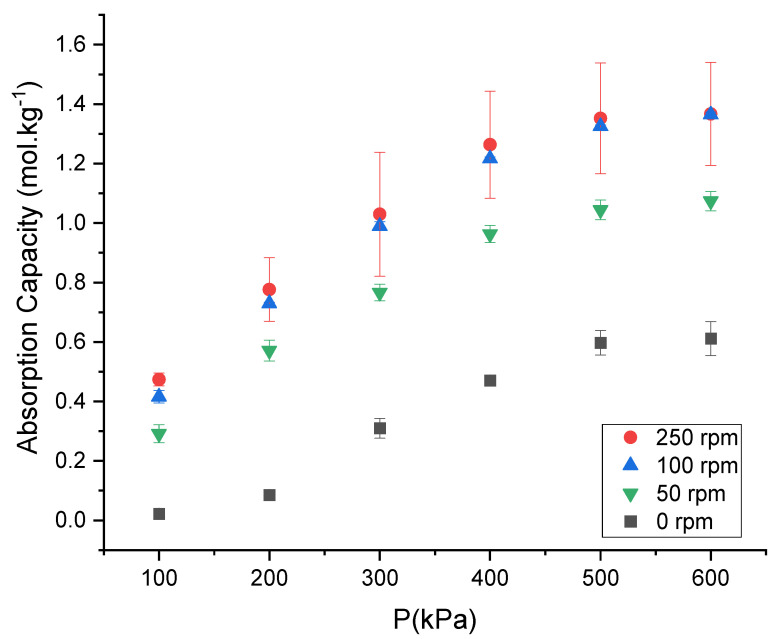
CO_2_ absorption capacity of ChCl:LvAc (1:2:0) at 0, 50, 100 and 250 rpm at 25 °C.

The CO_2_ absorption capacity of ChCl:LvAc (1:2:0) mole at 25 °C increases steadily with stirring speed as shown in Figure 5. The raw data are available in Table S3 in the supplementary information document.

At 6 bar and 25 °C, the CO_2_ absorption capacity of ChCl:LvAc (1:2:0) increased from 0.61 mol·kg^−1^ under stagnant conditions up to 1.37 mol·kg^−1^ under strong (max) stirring at 250 rpm. The CO_2_ absorption capacity increases strongly with increasing stirring speeds up to 100 rpm. An increase in stirring speed to a value beyond 100 rpm did not have a significant effect on the CO_2_ absorption capacity. Stirring can affect the CO_2_ absorption capacity of DESs in different ways. Firstly, a cylindrical beaker was used to hold the DES samples, and above a certain stirring speed, it was observed that the interface DES/gas suffered a gradual change from a circular shape (stagnant conditions) to a conical shape as the vortex in the fluid grew with increasing stirring speed. Hence, increments in stirring speed lead to larger surface areas for CO_2_ absorption when compared to stagnant conditions. Secondly, in the case of stagnant conditions, CO_2_ mass transfer occurs via the dissolution of CO_2_ at the surface of the DESs and via the transport of the CO_2_ molecules from the surface to the bulk of the liquid solely by molecular diffusion. When the DES is stirred, convection becomes a relevant factor and facilitates/boosts the transport of CO_2_ molecules from the interface to the bulk of the liquid. The facilitated mass transfer can be understood based on the two-film theory [18,19], and by understanding that stirring reduces the thickness of the stagnant liquid film that the CO_2_ molecules must cross by molecular diffusion before reaching well-mixed zones of the fluid.

The values of CO_2_ absorption capacity measured under stagnant conditions in this work are approximately similar to the ones reported by Ullah et al. at low pressures [15]. However, the values presented in this work are slightly higher at higher pressures, with the difference reaching approximately 0.16 mol·kg^−1^ at a pressure of 6 bar. Such difference should be at least partially due to differences in the equipment used.

### 3.4. Statistical Analysis of CO_2_ Absorption Results

The experimental design of experiments allows for the determination of the operating parameters at which the maximum absorption capacity could be achieved. Furthermore, the interactions among these parameters might give a deeper understanding of the CO_2_ absorption behaviour in ChCl:LvAc DESs.

**Figure 6:**
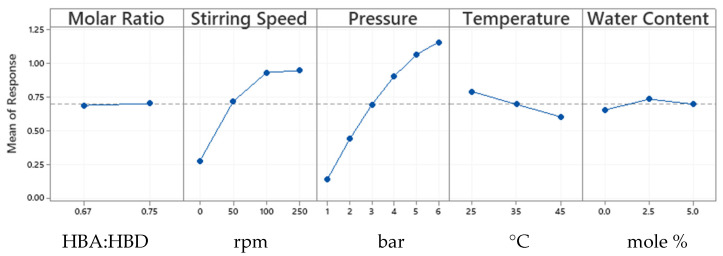
Main effects plot for CO_2_ absorption in ChCl:LvAc DESs.

All controlled factors have statistically relevant effects on the CO_2_ absorption in ChCl:LvAc DESs with *p*-values < 0.05. Figure 6 presents the main effects plot with the mean response for each factor. The pressure is the most significant factor with a contribution of 79.31% to the model describing CO_2_ absorption in ChCl:LvAc DESs, followed by the stirring speed with 14%, temperature with 2.8%, water content with 0.39% and finally the HBA:HBD molar ratio with a contribution of 0.79%. Table S4 in the supplementary information document provides additional information. We can conclude that CO_2_ absorption in ChCl:LvAc DESs increases strongly with increasing the pressure and stirring speed, decreases moderately with increasing temperature and is minimally affected by the HBA:HBD molar ratio and by the water content in the DES.

The second-order and higher interactions of factors have a very small contribution to the model. More details are available in the supplementary information document in Sections F–P.

### 3.5. The Effect of Water Content on CO_2_ Absorption

**Figure 7:**
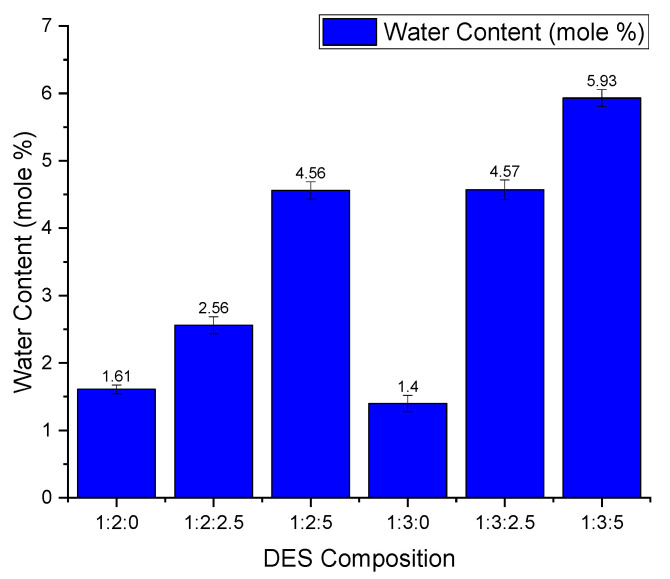
Water content (mole %) in different ChCl:LvAc DES compositions.

ChCl:LvAc DESs are highly hygroscopic, as reported by Delgado et al. [17] Therefore, even the DES compositions in which no water was added (i.e., 1:2:0 and 1:3:0) will always contain some residual water. Therefore, although the statistical analysis of the results shows the effect of each experimental parameter on the CO_2_ absorption in ChCl:LvAc DESs, the result obtained for the effect of the water content should be treated cautiously and deserves a more accurate analysis. Such analysis can only be carried out if the “real” water content of the DESs is accurately known, therefore, Karl Fisher titration was used for this purpose and the results are shown in Figure 7.

**Figure 8:**
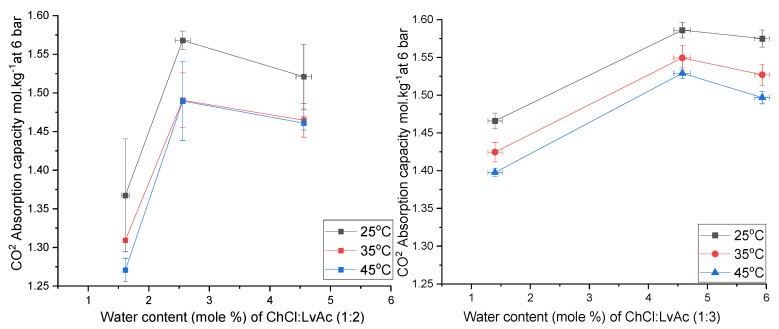
The effect of water content on the CO_2_ absorption capacity of ChCl:LvAc (1:2) (**left**) and (1:3) (**right**) at 6 bar and at different temperatures.

For both HBD:HBA molar ratios, the CO_2_ absorption capacity first increases with increasing water content but then decreases above a certain value of water content (see Figure 8). This effect is more pronounced for ChCl:LvAc (1:2) than for ChCl:LvAc (1:3). We hypothesise that the addition of some water reduces the viscosity of the DES, thus facilitating the diffusivity of CO_2_ molecules in the liquid film. However, adding excessive water reduces CO_2_ absorption due to the reduced CO_2_ solubility in water [13].

### 3.6. CO_2_ Desorption Results

#### 3.6.1. Indication of Nonspontaneous Released (Residual) CO_2_ by Weight Gain Measurements

**Figure 9:**
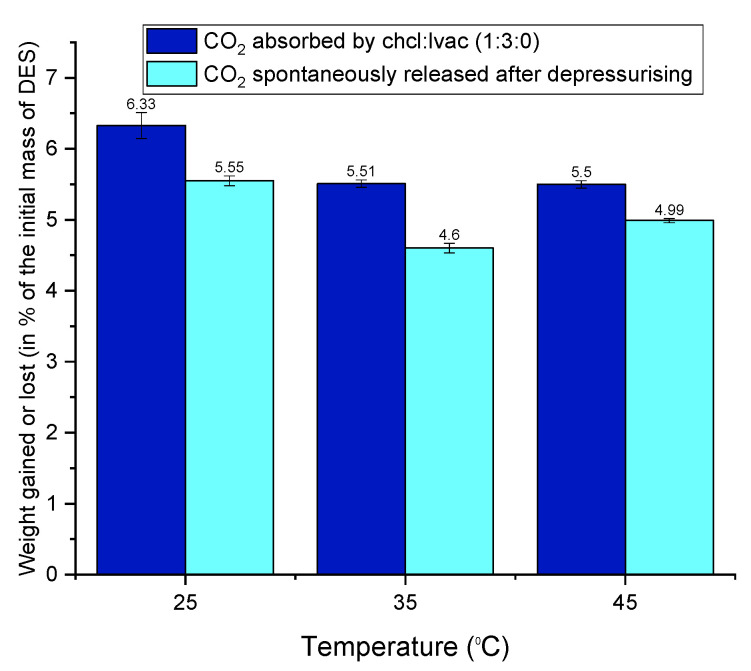
Weight gained by ChCl:LvAc (1:3:0) after CO_2_ absorption at 25 °C, 35 °C and 45 °C and lost by CO_2_ released spontaneously after depressurisation.

The absolute pressure drop in the system indicates the absorption of CO_2_ by the DES under investigation. The amount of CO_2_ absorbed is calculated using the ideal gas law as per Equation (2) above. The thermodynamics of the absorption process (discussed in Section 3.2) shows that CO_2_ absorption by the DESs is a nonspontaneous phenomenon. Therefore, the spontaneous release of most of the absorbed CO_2_ is expected (and indeed observed) once the VLE rig is depressurised. The weighing of the DES samples before and after the absorption test allows for the determination of the CO_2_ that remains in the DES after depressurisation (residual CO_2_) and must be released by other methods (e.g., heat treatment of the DESs). The difference between the CO_2_ content according to pressure drop calculations and the CO_2_ content after depressurising corresponds to the CO_2_ that is released spontaneously. As an example, this information is presented in Figure 9 for ChCl:LvAc (1:3:0).

Figure 9 shows that the absorption capacity of ChCl:LvAc DES (1:3:0) decreases with increasing temperature. Similar behaviour was observed for all the other ChCl:LvAc compositions.

#### 3.6.2. Quantitative Use of FTIR to Follow the Desorption of Nonspontaneous Released (Residual) CO_2_

**Figure 10:**
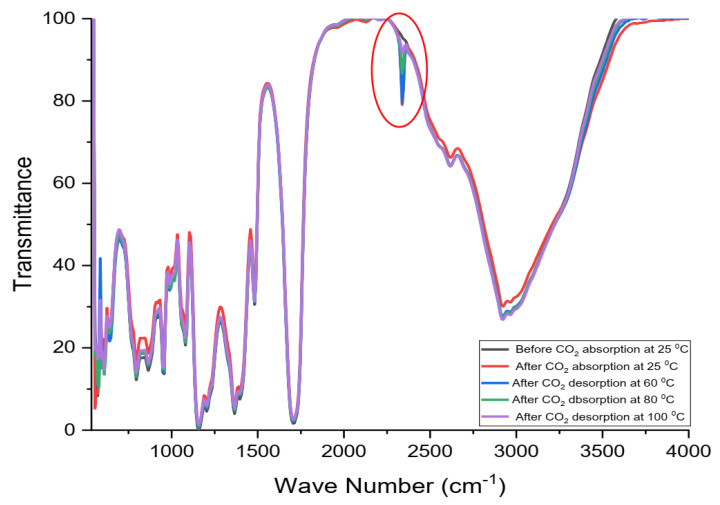
The FTIR spectra of ChCl:LvAc (1:3:0) before and after CO_2_ absorption at 25 °C and CO_2_ desorption at different temperatures.

Figure 10 displays the FTIR spectra of ChCl:LvAc (1:3:0) before and after CO_2_ absorption and desorption at different temperatures. The FTIR transmittance peak associated with the double carbonyl O=C=O functional group (visible at 2340 cm^−1^) was only detected after the CO_2_ absorption tests. Similar behaviour was observed for all the other ChCl:LvAc compositions.

Increasing the regeneration temperature results in a progressive release of CO_2_ from DES samples with residual CO_2_ as shown in Figure 10. The FTIR transmittance peak associated with the double carbonyl O=C=O functional group (≈2340 cm^−1^) decreases when the DES containing CO_2_ is heated at a temperature of 60 °C or above.

#### 3.6.3. Indication of CO_2_ Desorption by Weight Loss Measurements

**Figure 11:**
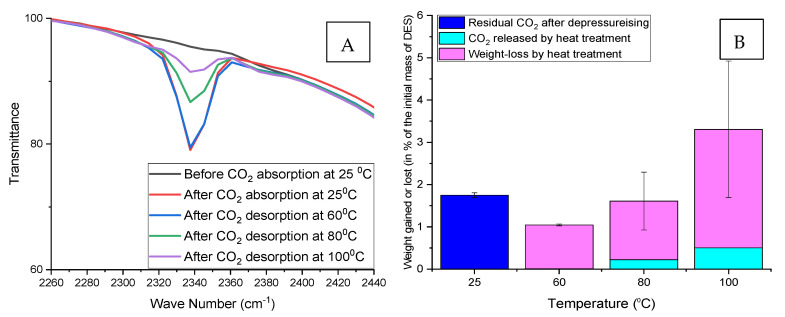
Regeneration of ChCl:LvAc (1:3:0) after CO_2_ absorption at 25 °C. (**A**) Reduction in FTIR transmittance peak associated with the double carbonyl group with increasing regeneration temperature; (**B**) residual CO_2_ after depressurisation, CO_2_ released by heat treatment and weight loss after regeneration at different temperatures.

**Figure 12:**
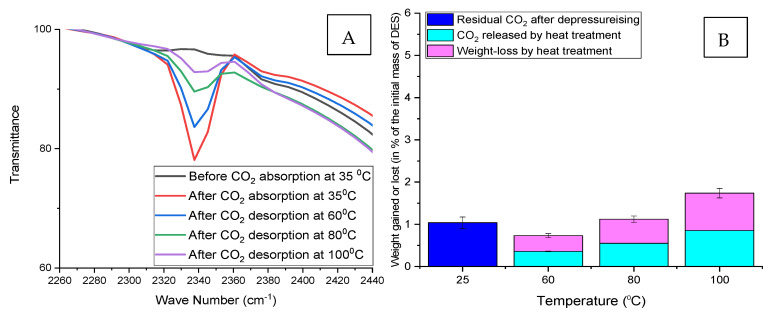
Regeneration of ChCl:LvAc (1:3:0) after CO_2_ absorption at 35 °C. (**A**) Reduction in FTIR transmittance peak associated with the double carbonyl group with increasing regeneration temperature; (**B**) residual CO_2_ after depressurisation, CO_2_ released by heat treatment and weight loss after regeneration at different temperatures.

**Figure 13:**
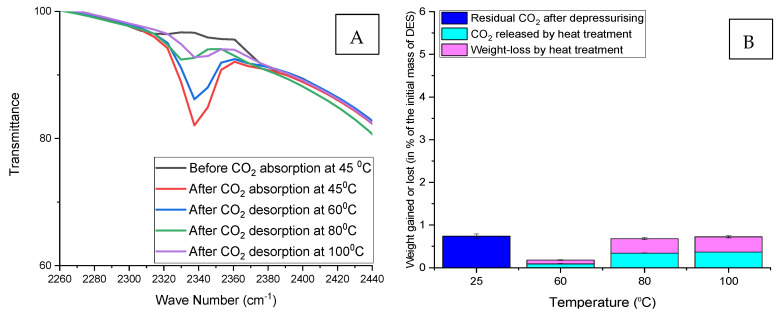
Regeneration of ChCl:LvAc (1:3:0) after CO_2_ absorption at 45 °C. (**A**) Reduction in FTIR transmittance peak associated with the double carbonyl group with increasing regeneration temperature; (**B**) residual CO_2_ after depressurisation, CO_2_ released by heat treatment and weight loss after regeneration at different temperatures.

The weight loss method was also used to verify the CO_2_ desorption at different temperatures. It also compares the weight gained by the DESs after CO_2_ absorption with the total weight reduction due to the heat treatment (regeneration) process. Figure 11, Figure 12 and Figure 13 below show that the weight of DES samples with residual CO_2_ decreases with increasing absorption and desorption temperatures simultaneously.

Weight loss due to the regeneration process is always higher than the weight of CO_2_ in the DES before the start of the regeneration (heat treatment) process. In short, the weight loss is not limited to CO_2_ desorption during the regeneration process but also due to the partial evaporation of the water contained in the DES.

### 3.7. Total CO_2_ Released

**Figure 14:**
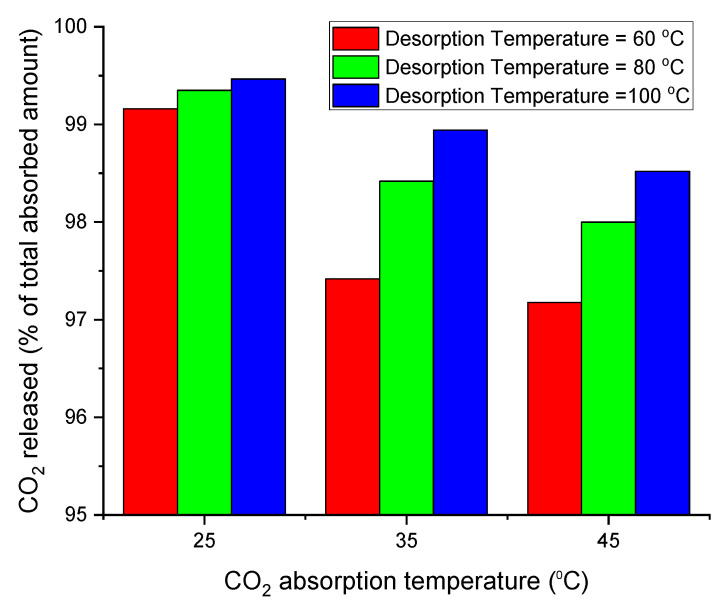
The total CO_2_ released from ChCl:LvAc (1:3:0) samples that absorbed CO_2_ at 25 °C, 35 °C and 45 °C and released CO_2_ at 60 °C, 80 °C and 100 °C.

The total amount of CO_2_ released from the DES sample is the combination of the CO_2_ amount released spontaneously after depressurisation and the amount released by heat treatment as per Figure 14 below. The regeneration temperature of 80 °C is the temperature at which most CO_2_ is released with a minimal reduction in DES weight according to weight loss measurements. Furthermore, at 80 °C, the transmittance peak associated with the double carbonyl group of the CO_2_ molecule is minimal, indicating that most of the initial CO_2_ was released. because of these two reasons, 80 °C might be considered as the optimal regeneration temperature for ChCl:LvAc DESs.

### 3.8. Recyclability of the DESs

**Figure 15:**
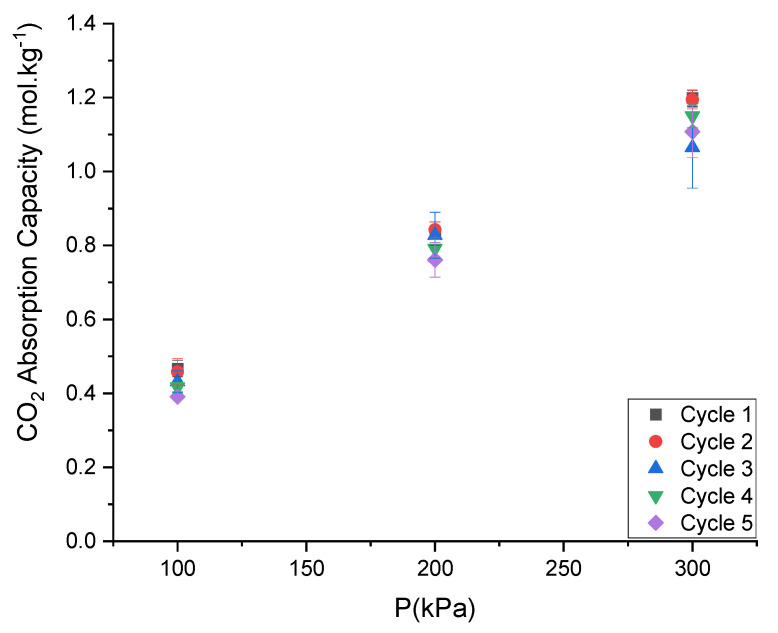
Variation of the CO_2_ absorption capacity of ChCl:LvAc (1:2:0) over five consecutive absorption/desorption cycles: values measured at 25 °C and 250 rpm.

The CO_2_ absorption capacity of ChCl:LvAc (1:2:0) slightly decreases with the recycling of the sorbent at 80 °C and 250 rpm as shown in Figure 15. 

The CO_2_ absorption capacity of ChCl:LvAc (1:2:0) slightly decreased from 1.2 mol·kg^−1^ in the first absorption cycle to 1.11 mol·kg^−1^ in the fifth cycle, at the same operating conditions (25 °C, 3 bar and stirring rate of 250 rpm).

**Figure 16:**
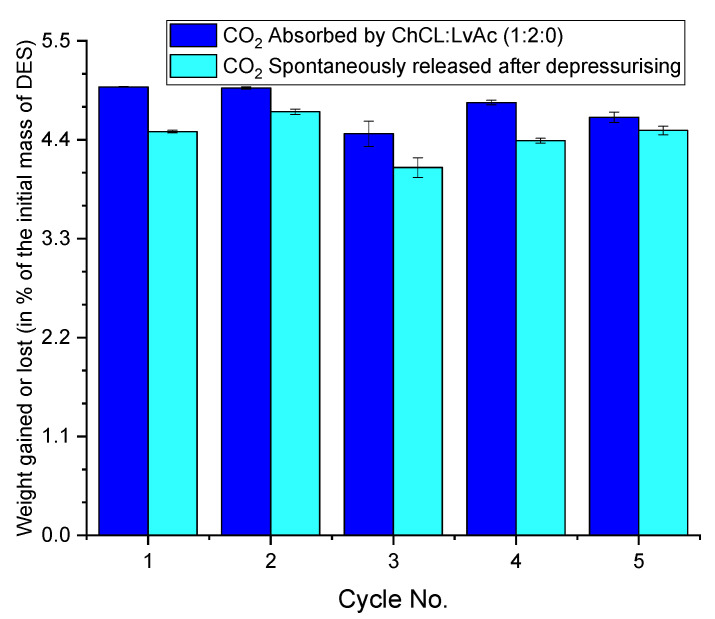
Weight gained through CO_2_ absorption for 5 cycles at 25 °C and CO_2_ released spontaneously after depressurisation. Experiments carried out with ChCl:LvAc (1:2:0) at 25 °C and 250 rpm.

Figure 16 shows that the CO_2_ absorption capacity of ChCl:LvAc (1:2:0) slightly decreases with reusing the sorbent. It also shows that, for the same DES composition and operating conditions, the amount of CO_2_ that is released spontaneously after the depressurisation of the system can vary from cycle to cycle.

One of the major disadvantages of utilising the MEA for carbon capture processes is the huge loss of the sorbent during the regeneration process due to the formation of carbamates during the absorption process [38]. Additionally, the regeneration of the MEA is costly as it must be performed at high temperatures [40,41]. On the other hand, in this work DESs samples have lost only 0.48% of their initial weight after five consecutive cycles of CO_2_ absorption at 25 °C and desorption/regeneration at 80 °C.

### 3.9. Selectivity of the DESs towards CO_2_

**Figure 17:**
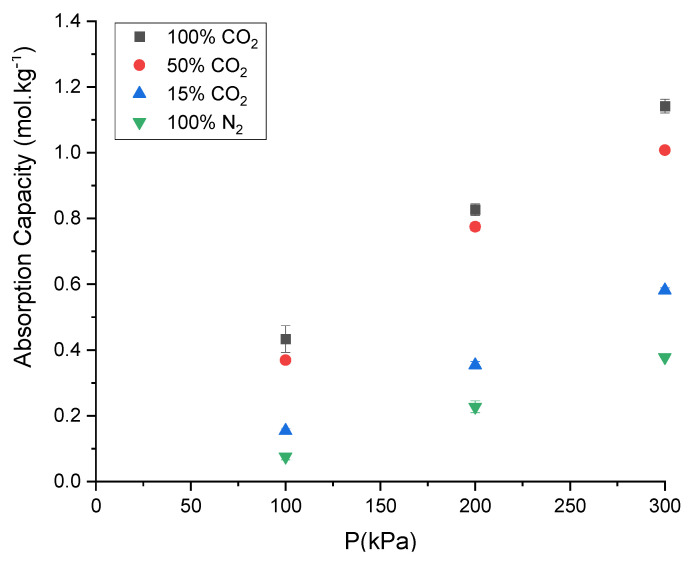
Absorption capacity of ChCl:LvAc (1:3:2.5) for different gas mixtures of CO_2_ and N_2_. Experiments carried out at 25 °C and 250 rpm.

The CO_2_ absorption capacity of ChCl:LvAc (1:3:2.5) decreases with decreasing the molar ratio of CO_2_ in the gas phase at 25 °C and 250 rpm as shown in Figure 17.

**Figure 18:**
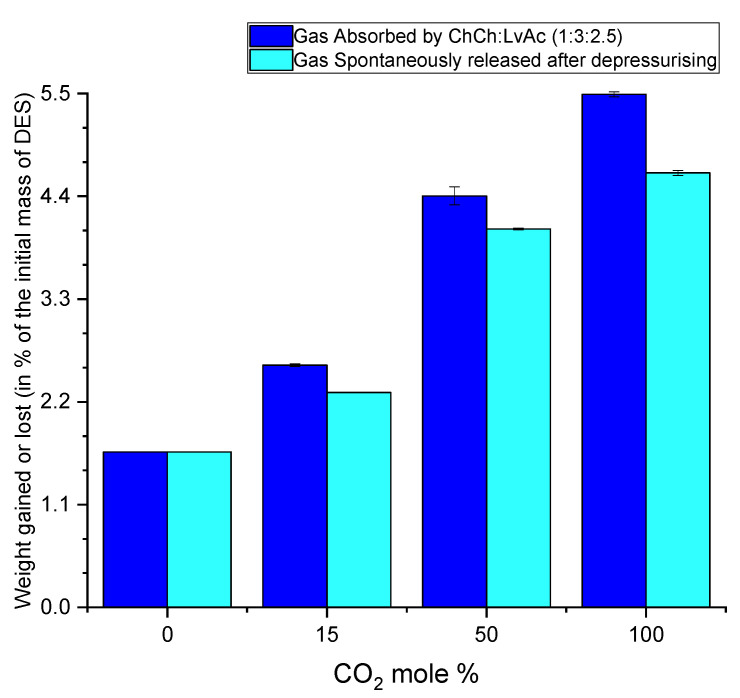
Weight gained by ChCl:LvAc (1:3:2.5) after gas absorption at 25 °C and 250 rpm, and lost by the spontaneous release of gas after depressurisation.

The absorption capacity of ChCl:LvAc (1:3:2.5) at 3 bar decreases from 1.14 mol·kg^−1^ for pure CO_2_ to 1.0 mol·kg^−1^ for gas mixtures with 50% CO_2,_ and finally to 0.58 mol·kg^−1^ for gas mixtures with 15% CO_2_. The reduction in the absorption capacity of ChCl:LvAc (1:3:2.5) is proportional to the molar fraction of the CO_2_ in the (feed) gas mixtures as per Figure 18 and as reported by Blauwhoff et al. [42] Nitrogen has more limited solubility in the DES (0.37 mol·kg^−1^) under the same conditions and is all completely released from the DES after depressurisation.

**Figure 19:**
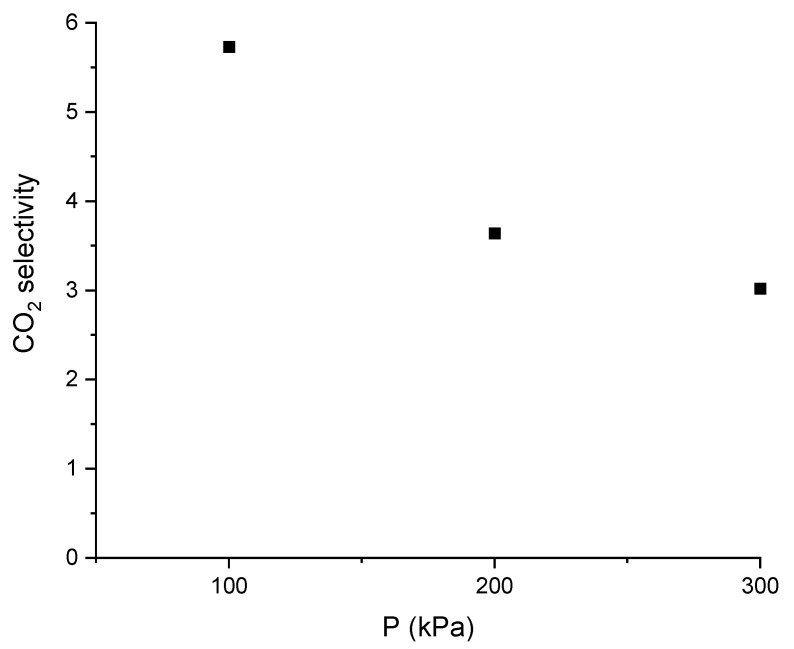
CO_2_ selectivity based on the absorption of pure gases by ChCl:LvAc (1:3:2.5) at 25 °C and 250 rpm.

On the other hand, ChCl:LvAc (1:3:2.5) seems to be more selective towards CO_2_ over N_2_ at lower pressures as seen in Figure 19. The selectivity towards CO_2_ seems to decline asymptotically as pressure increases.

The selectivity towards CO_2_ in a mixture of gases in ChCl:LvAc (1:3:2.5) was calculated and compared to the selectivity of ionic liquids taken from the literature (see Table 3). Compared to ionic liquids, the ChCl:LvAc DES exhibits lower CO_2_/N_2_ selectivities.

## 4. Conclusions

Choline-chloride:levulinic-acid-based DESs were prepared successfully at different molar ratios and water contents. The CO_2_ absorption in ChCl:LvAc DESs was obtained at different molar ratios, pressures, water content, stirring speeds and temperatures. Thermodynamic properties (∆H, ∆S and ∆G) of CO_2_ absorption in ChCl:LvAc DESs showed that CO_2_ absorption is exothermic and nonspontaneous.

In this study, FTIR spectrophotometry was utilised as a secondary method to identify the absorption and desorption of CO_2_ by ChCl:LvAc DESs at different conditions of temperature pressures. The FTIR transmittance peak associated with the double carbonyl O=C=O functional group (visible at 2340 cm^−1^) was only detected after the CO_2_ absorption tests, while this transmittance peak decreases with increasing DES regeneration temperature.

The regeneration process at 80 °C is a temperature at which most CO_2_ is released with a minimal reduction in DES weight according to weight loss measurements. The residual amount of CO_2_ in the DES that cannot be released by heat treatment was calculated using the Beer–Lambert law.

All the main input factors (pressure, stirring speed, temperature, water content and molar ratio) have statistical significance on the CO_2_ absorption by ChCl:LvAc DESs. The pressure is found to be the most significant factor, followed by stirring speed, temperature and finally water content and HBA:HBD molar ratio.

In this work, ChCl:LvAc DESs exhibited good recyclability as samples lost only 0.48% of their initial weight after five consecutive cycles of CO_2_ absorption at 25 °C and desorption at 80 °C. Finally, ChCl:LvAc DESs showed moderate selectivity towards CO_2_ over N_2_, with up to 5.63 at 50% CO_2_.

## Figures and Tables

**Table 1:** Partial pressures of CO_2_ and N_2_ and the total pressure applied in the selectivity tests.

Run	Before Measurements: Pressure in Buffer Tank Only (During Temperature Equilibration)		At the Start of the Measurements: Pressure in the Buffer and Measuring Tanks	
	p_CO_2__ (bar)	p_N_2__ (bar)	p_CO_2__ (bar)	p_N_2__ (bar)
Pure CO_2_	6	0	3	0
50% CO_2_/50% N_2_	3	3	1.5	1.5
15% CO_2_/85% N_2_	0.9	5.1	0.45	2.55
Pure N_2_	0	6	0	3

**Table 2:** Calculated values of Henry’s law constant, enthalpy change, entropy change and change in Gibbs free energy of CO_2_−DESs at 1 bar.

CO_2_−DESs	T (K)	Hx (MPa)	∆H (kJ·mole^−1^)	∆S (J·mole^−1^·K^−1^)	∆G (kJ·mole^−1^)
ChCl:LvAc (1:2:0)	298.15	5.34	−41.5	−172.4	9.86
	308.15	6.92	−49.6	−196.1	10.9
	318.15	12.1	−65.9	−246.8	12.7
ChCl:LvAc (1:2:2.5)	298.15	6.14	−45.0	−185.2	10.2
	308.15	8.88	−56.0	−218.9	11.5
	318.15	10.5	−62.2	−234.4	12.3
ChCl:LvAc (1:2:5)	298.15	5.47	−42.1	−174.6	9.92
	308.15	8.17	−53.8	−211.3	11.3
	318.15	10.3	−61.8	−232.8	12.3
ChCl:LvAc (1:3:0)	298.15	5.60	−42.7	−176.8	9.97
	308.15	10.1	−59.3	−230.7	11.8
	318.15	10.8	−63.0	−237.1	12.4
ChCl:LvAc (1:3:2.5)	298.15	4.61	−37.9	−159.0	9.49
	308.15	9.25	−57.0	−222.6	11.6
	318.15	10.8	−63.0	−237.1	12.4
ChCl:LvAc (1:3:5)	298.15	4.96	−39.7	−165.7	9.67
	308.15	9.29	−57.1	−223.0	11.6
	318.15	14.2	−70.3	−262.0	13.1

**Table 3:** Selectivity of some absorbents at 100 kPa and 25 °C.

Material	Description	Molar % of Gases (CO_2_/N_2_)	Selectivity (S)	Reference
DES	ChCl:LvAc (1:3:2.5)	50/50	5.63	This work
		15/85	4.94	
IL	bmim (BF4)		28.7 *	[43]
IL	bmim (BF6)		22.6 *	[44]

* selectivity values were calculated based on pure components; measurements were done at 30 °C.

## Data Availability

The data presented in this study are available on request from the corresponding author.

## Supplementary Materials

The following are available online. Figures S1–S13, Tables S1–S5, Equations (S1)–(S9).

## Sample Availability

Samples of the compounds are not available from the authors.
