# Supplementary material for: A Comprehensive Study of CO2 Absorption and Desorption by Choline-Chloride/Levulinic-Acid-Based Deep Eutectic Solvents
Source: Molecules. 2021 Sep 15;26(18):5595. doi: 10.3390/molecules26185595 (PMC8465201; doi:10.3390/molecules26185595)
Supplement: Supplementary file 1 [file molecules-26-05595-s001.zip › molecules-1344506-supplementary.pdf]

A comprehensive study of CO_2 absorption and desorption by choline chloride levulinic acid-based deep eutectic solvents

Mohaned Aboshatta, Vitor Magueijo
Department of Chemical and Process Engineering, University of Strathclyde, Glasgow, UK
Mohaned.aboshatta@strath.ac.uk

Supplementary Information Document

1. Determination of baselines

1.1 Baselines VS. absorption measurements at 295.15 K of ChCl: LvAc (1:3:0)

The VLE rig reaches equilibrium at 298.15 K within 2 hours as shown in below. The absolute pressure drop due to the CO_2 absorption only is the difference between the two isotherms pressure drop curves in fig.S1 below.

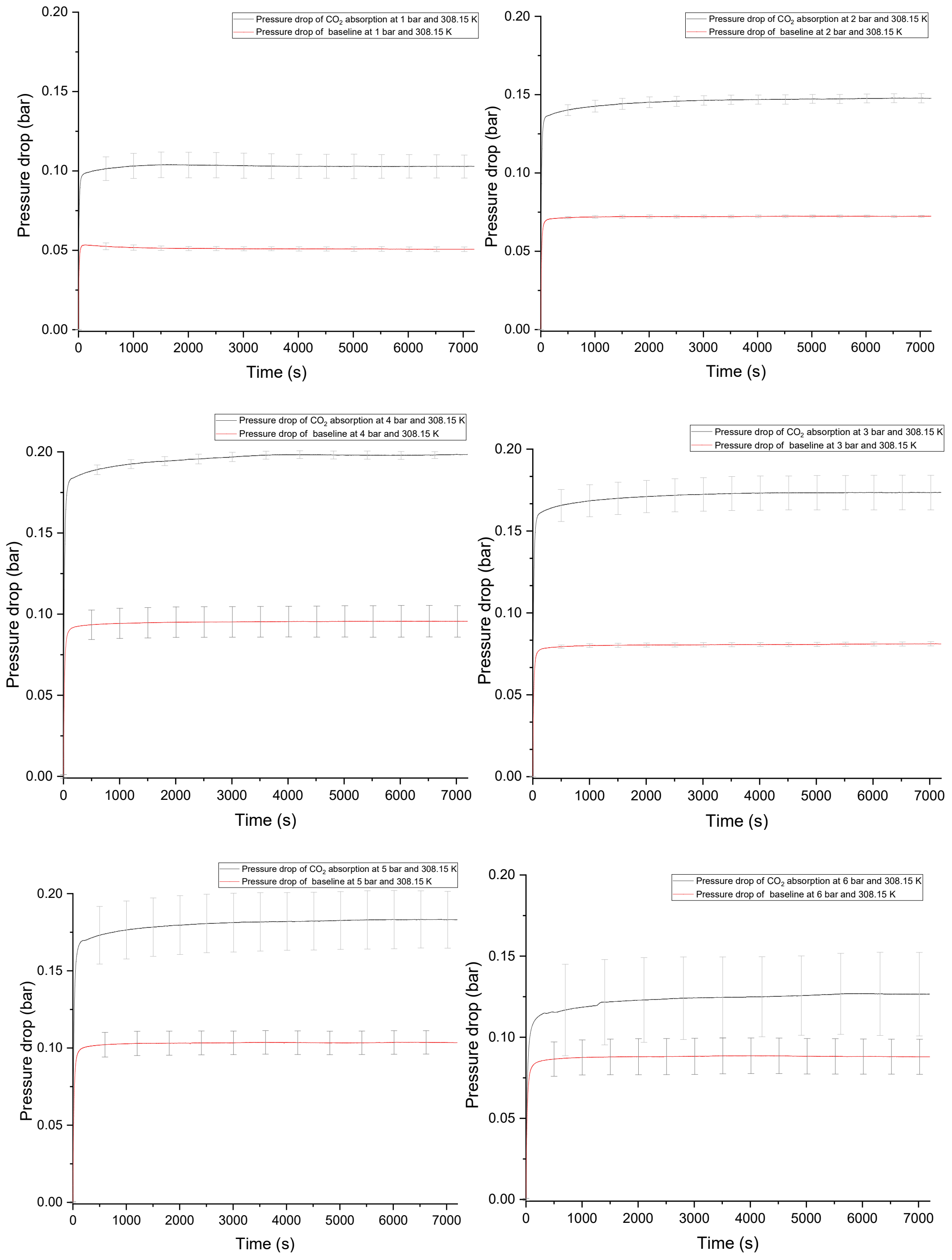

Figure S1. Pressure drop values of the absolute pressure drop in the rig due to CO_2 absorption by ChCl: LvAc (1:3:0) at 308.15 K Vs. pressure drop at the baselines

1.2 Baselines VS. absorption measurements at 318.15 K of ChCl: LvAc (1:3:0)

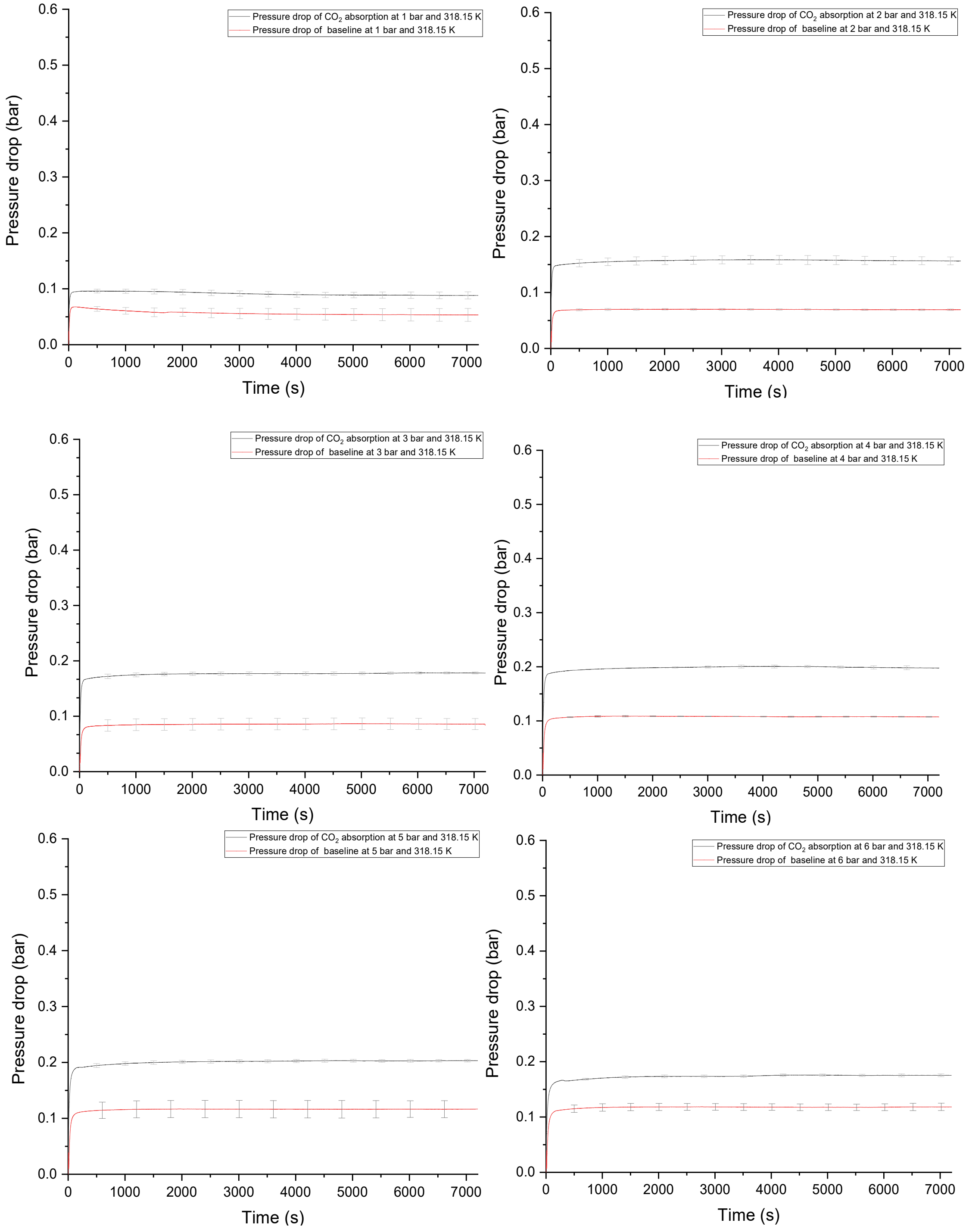

Figure S2. Pressure drop values of the absolute pressure drop in the rig due to CO_2 absorption by ChCl: LvAc (1:3:0) at 318.15 K Vs. pressure drop at the baselines

2. Calibration of the VLE rig using Monoethanolamine at 45 °C

A. Thermodynamic Equilibrium

The VLE rig reaches equilibrium within 2 hours as shown in fig.S3 below, the pressure drop in the system decreases with increasing the pressure at a constant temperature of 318.15 K

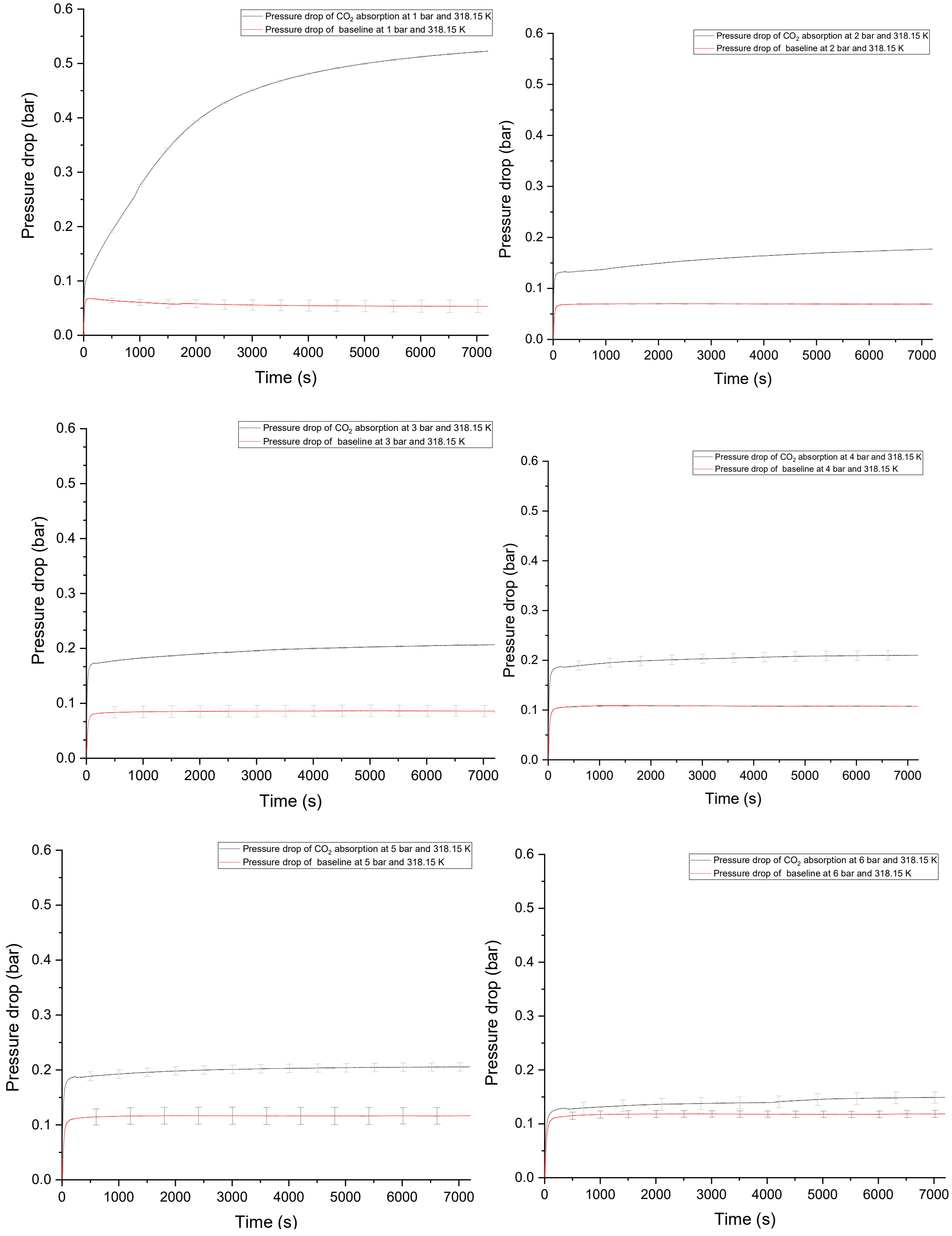

Figure S3. Pressure drop values of the absolute pressure drop in the rig due to CO_2 absorption by MEA Vs. pressure drop at the baselines

B. CO_2 load calculation

CO_2 load increases with increasing the pressure in the system as extensively reported in the literature (1). Lee et al. in their work in (1) reported the CO_2 load absorbed by MEA (30% wt.) at pressures ranging between 0.1 – 1000 kPa as shown in table 1 and fig. 2 below. However, we have estimated CO_2 load absorbed by MEA (30% wt.) at pressures of 100, 200, 300, 400, 500 and 600 kPa at 318.15 K by fitting a nonlinear power-law fit to the raw data reported by Lee et al. (1). The fitted model is highly precise of R^2 value of 100%. Then, we compared our calculated values against estimated values at certain pressures as per fig. S4 below:

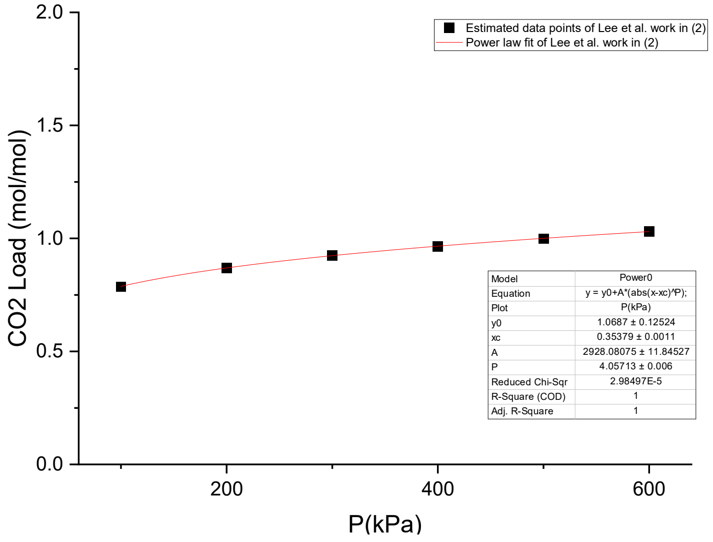

Figure S4. CO_2 load at 318.15 from the estimated data from Lee et al. work in (1) with a nonlinear fitting

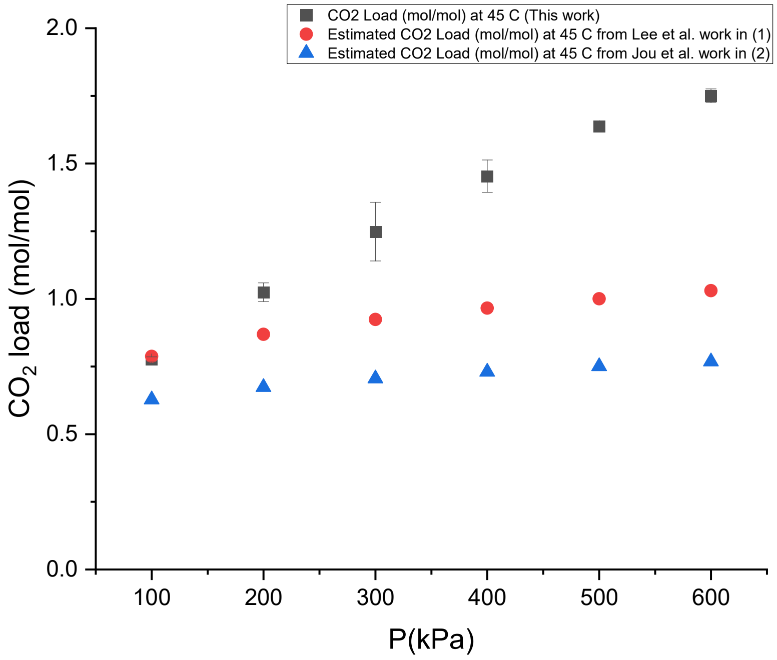

Figure S5. CO_2 load at 318.15 K of this work Vs. Lee et al. work in (1) and Jou et a.l work in (2)

Table S1. Calculated correction factors at 318.15 K

Estimated values of CO_2 Load at given pressures from using nonlinear fitting from Lee et al. work in (1)		This work		Correction Factor (CF)
CO2 Load (mol/mol) at 318.15 K	P(kPa)	CO2 Load (mol/mol) at 318.15 K	P(kPa)	
0.7877	100	0.7759	100	1.0152
0.8692	200	1.0247	200	0.8482
0.9236	300	1.2484	300	0.7398
0.9656	400	1.4535	400	0.6643
1.0003	500	1.6384	500	0.6105
1.0301	600	1.7504	600	0.5885

C. Correction factors

Fig.S4 above shows a clear deviation between the calculated values of CO_2 load in MEA (30% wt.) at 318.15 from the rig equilibrium data and the ones reported in the literature under the same conditions. Therefore, correction factors (CFs) must be estimated and applied to compensate for the deviation. CF is applicable only for systematic errors, hence to ensure that the error is a systematic one we need to check how calculated values gained from the rig fit with the nonlinear power-law model that fitted with the raw data from the literature (1) as per fig.S4 above. The calculated values fit well with the nonlinear model of R^2 value of 99.77% as per fig.4 below. Therefore, it is justifiable to generate a linear CFs between gained results and the ones reported in the literature as per table S1 below.

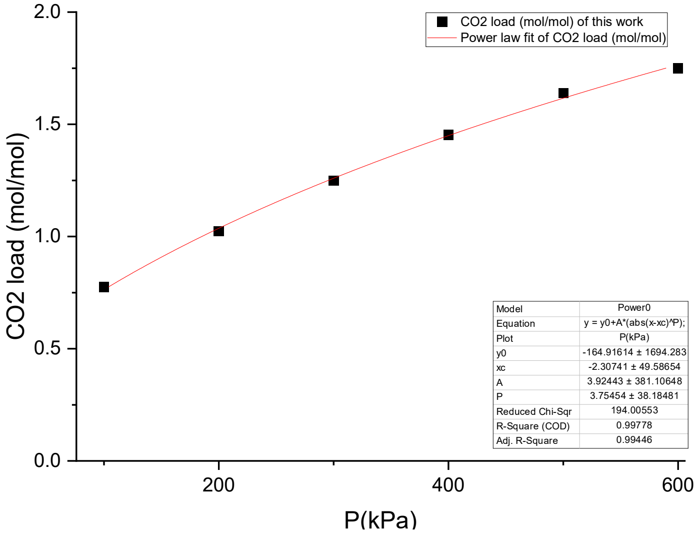

Figure S6. Power-law fitting CO_2 load at 318.15 of this work

Table S2. Factors and levels of the factorial design at different temperatures

Molar ratio	Water content	Temperature °C	Pressure (bar)	CO_2 Absorption (mol.kg^{-1})
1:2	0	25	1	0.4741
1:2	0	25	2	0.7767
1:2	0	25	3	1.02991
1:2	0	25	4	1.26366
1:2	0	25	5	1.3525
1:2	0	25	6	1.36739
1:2	2.5	25	1	0.36352
1:2	2.5	25	2	0.69315
1:2	2.5	25	3	0.9926
1:2	2.5	25	4	1.26322
1:2	2.5	25	5	1.43897
1:2	2.5	25	6	1.56806
1:2	5	25	1	0.45091
1:2	5	25	2	0.76693
1:2	5	25	3	1.09265
1:2	5	25	4	1.37936
1:2	5	25	5	1.50813
1:2	5	25	6	1.52095
1:3	0	25	1	0.38898
1:3	0	25	2	0.78817
1:3	0	25	3	1.0001
1:3	0	25	4	1.25297
1:3	0	25	5	1.44295
1:3	0	25	6	1.46589
1:3	2.5	25	1	0.43321
1:3	2.5	25	2	0.82708
1:3	2.5	25	3	1.14201
1:3	2.5	25	4	1.43815
1:3	2.5	25	5	1.46695
1:3	2.5	25	6	1.58618
1:3	5	25	1	0.40692
1:3	5	25	2	0.71202
1:3	5	25	3	1.01957
1:3	5	25	4	1.28441
1:3	5	25	5	1.43618
1:3	5	25	6	1.57494
1:2	0	35	1	0.2856
1:2	0	35	2	0.73661
1:2	0	35	3	0.99646
1:2	0	35	4	1.12232
1:2	0	35	5	1.28709

Molar ratio	Water content	Temperature °C	Pressure (bar)	CO_2 Absorption (mol.kg^{-1})
1:2	0	35	6	1.30934
1:2	2.5	35	1	0.25299
1:2	2.5	35	2	0.65864
1:2	2.5	35	3	0.91296
1:2	2.5	35	4	1.09089
1:2	2.5	35	5	1.29432
1:2	2.5	35	6	1.49044
1:2	5	35	1	0.28944
1:2	5	35	2	0.71323
1:2	5	35	3	0.98762
1:2	5	35	4	1.18212
1:2	5	35	5	1.36429
1:2	5	35	6	1.46463
1:3	0	35	1	0.25059
1:3	0	35	2	0.70921
1:3	0	35	3	0.9976
1:3	0	35	4	1.19119
1:3	0	35	5	1.35511
1:3	0	35	6	1.42458
1:3	2.5	35	1	0.23812
1:3	2.5	35	2	0.66805
1:3	2.5	35	3	0.99417
1:3	2.5	35	4	1.19821
1:3	2.5	35	5	1.40254
1:3	2.5	35	6	1.54939
1:3	5	35	1	0.25323
1:3	5	35	2	0.70565
1:3	5	35	3	1.0143
1:3	5	35	4	1.23403
1:3	5	35	5	1.41172
1:3	5	35	6	1.52696
1:2	0	45	1	0.20586
1:2	0	45	2	0.55889
1:2	0	45	3	0.83784
1:2	0	45	4	1.05581
1:2	0	45	5	1.25663
1:2	0	45	6	1.27078
1:2	2.5	45	1	0.22173
1:2	2.5	45	2	0.53081
1:2	2.5	45	3	0.79991
1:2	2.5	45	4	1.02709
1:2	2.5	45	5	1.24891
1:2	2.5	45	6	1.48952

Molar ratio	Water content	Temperature °C	Pressure (bar)	CO_2 Absorption (mol.kg^{-1})
1:2	5	45	1	0.19792
1:2	5	45	2	0.5431
1:2	5	45	3	0.83104
1:2	5	45	4	1.06314
1:2	5	45	5	1.29621
1:2	5	45	6	1.46098
1:3	0	45	1	0.17272
1:3	0	45	2	0.47796
1:3	0	45	3	0.7596
1:3	0	45	4	1.00449
1:3	0	45	5	1.24484
1:3	0	45	6	1.39762
1:3	2.5	45	1	0.20866
1:3	2.5	45	2	0.5039
1:3	2.5	45	3	0.84396
1:3	2.5	45	4	1.17127
1:3	2.5	45	5	1.47713
1:3	2.5	45	6	1.52899
1:3	5	45	1	0.15801
1:3	5	45	2	0.50721
1:3	5	45	3	0.81658
1:3	5	45	4	1.02373
1:3	5	45	5	1.27446
1:3	5	45	6	1.49683

D. The effect of stirring speed on the CO_2 absorption capacity

Table S3. The effect of stirring speed on CO_2 absorption capacity of ChCl:LvAc (1:2:0) at 298.15 K

Pressure (kPa)	Stirring Speed (rpm)			
	0	50	100	250
100	0.02295	0.29192	0.41624	0.4741
200	0.08609	0.57144	0.73008	0.7767
300	0.31007	0.76677	0.98967	1.02991
400	0.47134	0.96334	1.21714	1.26366
500	0.59781	1.04431	1.32556	1.3525
600	0.61153	1.07413	1.36501	1.36739

E. Thermodynamics analysis of CO_2 absorption

i. Henry's law constant (Hx)

The solubility of CO_2 in the DESs could be expressed in terms of HLC. The smaller the value of the HLC the higher the solubility (3), (4) and (5). H_x is defined as HLC based on mass fraction, could be expressed in equation S1 as follows

$$H_x(\mathrm{T,P}) = \lim_{\mathrm{x_{CO2}}\to 0} \mathrm{f}^{\mathrm{Liq}}_{CO_2} \left[\frac{(\mathrm{T, P, x_{CO2}})}{\mathrm{x_{CO2}}}\right] \qquad \text{Equation (S1)}$$

Where:

H_x (T, P): Henry's constant based on the mass fraction

$\mathrm{x_{CO2}}$: mass fraction of CO_2 in DES

c$f^{\mathrm{Liq}}_{CO_2}$ (T, P, X_{CO2}): fugacity of CO_2 in the DESs

When vapour-liquid equilibrium reached, the fugacity of CO_2 in the vapour phase is equal to that in the vapour phase as per S2 below, hence:

$$\mathrm{f}^{\mathrm{Liq}}_{CO_2}(\mathrm{T,P},x_{CO_2}) = \mathrm{f}^{\mathrm{Vap}}_{CO_2}(\mathrm{T,P},y_{CO_2}) = y_{CO_2}\mathrm{P}\ \phi_{CO_2}(\mathrm{T,P},y_{CO_2}) \qquad \text{Equation (S2)}$$

Where:

$f^{\mathrm{Vap}}_{CO_2}$ (T, P, y_{CO2}): fugacity of CO_2 in the vapour phase

y_{CO_2}: mole fraction of CO_2

ϕ_{CO2}: fugacity coefficient of CO_2 in the vapour phase, could be calculated using the three-term virial equation as per (6). Since the vapour pressure of the DESs is very small as compared to the vapour pressure of CO_2 (7). Hence, the vapour phase is composed of pure CO_2. In this case, Henry's law constant based on the mass fraction (Hx) can be expressed as per equations Equation (& S4 below:

$$H_x(\mathrm{T,P}) = \lim_{\mathrm{x_{CO2}}\to 0} \mathrm{f}\left[\frac{(\mathrm{T, P, x_{CO2}})}{\mathrm{x_{CO2}}}\right] = \lim_{\mathrm{x_{CO2}}\to 0} \mathrm{f}\left[\frac{(\mathrm{T, P, y_{CO2}})}{\mathrm{y_{CO2}}}\right] \qquad \text{Equation (S3)}$$

$$H_x(\mathrm{T,P}) = \frac{\mathrm{P}\ \phi_{\mathrm{CO2}}\ (\mathrm{T,P})}{\mathrm{x_{CO2}}} \qquad \text{Equation (S4)}$$

ii. Changes in enthalpy (ΔH), entropy (ΔS) and Gibbs free energy (ΔG)

Changes in enthalpy, Gibbs energy and entropy can be calculated from using Van't Hoff equations S5, S6, & S7 below:

$$\Delta \mathrm{G} = \mathrm{R\ TLn}\left[\frac{H(\mathrm{T,P})}{\mathrm{P0}}\right] \qquad \text{Equation (S5)}$$

$$\Delta \mathrm{H} = -\mathrm{R}\left\{\frac{\delta \mathrm{Ln}\left[\frac{H(\mathrm{T,P})}{\mathrm{P0}}\right]}{\delta\left(\frac{1}{\mathrm{T}}\right)}\right\} \qquad \text{Equation (S6)}$$

$$\Delta S = \left[\frac{\Delta H - \Delta G}{T}\right] \quad \text{Equation (S7)}$$

Where: P0 = 1 bar

F. Model equation

The full factorial design was analyzed by considering three factors and their interactions that forming the following model equation S8 :

$$Y = \beta_0 + \beta_1 x_1 + \beta_2 x_2 + \beta_3 x_3 + \beta_4 x_4 + \beta_5 x_5 + \beta_{12} x_1 x_2 + \beta_{13} x_1 x_3 + \cdots + \varepsilon \quad \text{Equation (S8)}$$

Where Y is the response (CO_2 absorption capacity), the β's are parameters whose values are to be determined, x_i represents the factors molar ratio, stirring speed, pressure, temperature and water content in the uncoded form and ε is a random error term. The developed model found to be highly accurate ($R^2 > 99\%$).

G. Analysis of variance

Analysis of variance of the full factorial design - table S4 in the supplementary information document- shows the effect of each controlled parameter (molar ratio, stirring speed, water content, pressure and temperature) and the second and third-order interactions among the parameters on the response (CO_2 absorption capacity).

As can be seen from table S4, all controlled parameters found to be significant (with P-value < 0.05), some of the second and interactions among the factors found to be significant as will be demonstrated in the following sections. Only one third-order interaction among the parameters found to be significant in this model equation. Coefficients of the parameters (the β's) of the model are presented in table S4.

H. Refining the model

Figure S7 in the supplementary information document reveals many insignificant interactions -below 2 - of different orders, therefore; it is essential to remove all the insignificant interactions from the model as per figure S7. The refined model equation – equation S1– is available in the supplementary information document whereas, the refined model summary is presented the refined model summary in **Error! Reference source not found.** below

I. Analysis of variance of the factorial designs

Table S4. Analysis of variance

Analysis of Variance for Transformed Response

Source	DF	Seq SS	Contribution	Adj SS	Adj MS
Model	55	3.34196	99.86%	3.34196	0.060763
Linear	13	3.25625	97.29%	1.04282	0.080217
Molar Ratio	1	0.02646	0.79%	0.00015	0.000148
Stirring Speed	3	0.46903	14.01%	0.37933	0.126445
Pressure	5	2.65425	79.31%	0.56519	0.113038
Temperature	2	0.09330	2.79%	0.09330	0.046650
Water Content	2	0.01320	0.39%	0.01320	0.006601
2-Way Interactions	38	0.08477	2.53%	0.08477	0.002231
Molar Ratio*Pressure	5	0.00436	0.13%	0.00360	0.000720
Molar Ratio*Temperature	2	0.00051	0.02%	0.00051	0.000254
Molar Ratio*Water Content	2	0.00016	0.00%	0.00016	0.000080
Stirring Speed*Pressure	15	0.03490	1.04%	0.03904	0.002603
Pressure*Temperature	10	0.04419	1.32%	0.04419	0.004419
Temperature*Water Content	4	0.00065	0.02%	0.00065	0.000162
3-Way Interactions	4	0.00095	0.03%	0.00095	0.000237
Molar Ratio*Temperature*Water Content	4	0.00095	0.03%	0.00095	0.000237
Error	70	0.00485	0.14%	0.00485	0.000069
Total	125	3.34681	100.00%		

Source	F-Value	P-Value
Model	877.48	0.000
Linear	1158.41	0.000
Molar Ratio	2.13	0.149
Stirring Speed	1825.99	0.000
Pressure	1632.37	0.000
Temperature	673.67	0.000
Water Content	95.33	0.000
2-Way Interactions	32.21	0.000
Molar Ratio*Pressure	10.40	0.000
Molar Ratio*Temperature	3.66	0.031
Molar Ratio*Water Content	1.15	0.323
Stirring Speed*Pressure	37.58	0.000
Pressure*Temperature	63.82	0.000
Temperature*Water Content	2.33	0.064
3-Way Interactions	3.42	0.013
Molar Ratio*Temperature*Water Content	3.42	0.013
Error		
Total		

J. Pareto charts of standardized and refined models

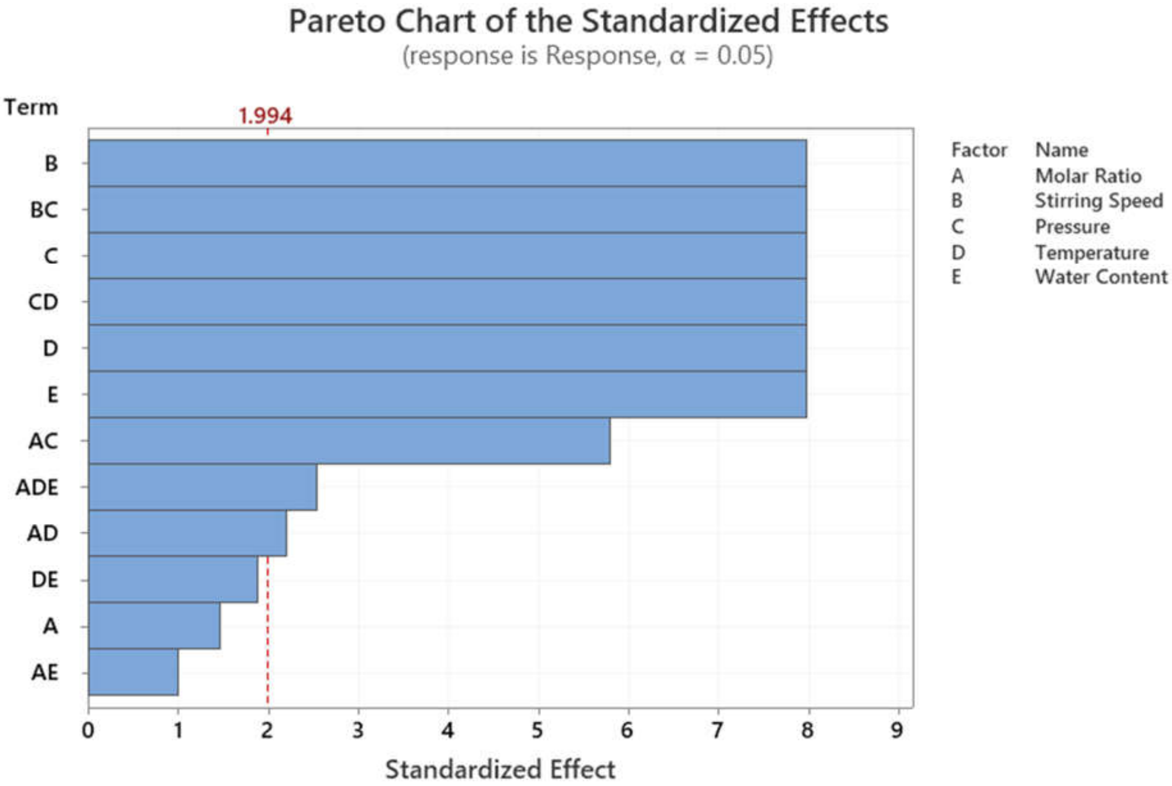

Figure S7. Pareto chart of the standardized effects

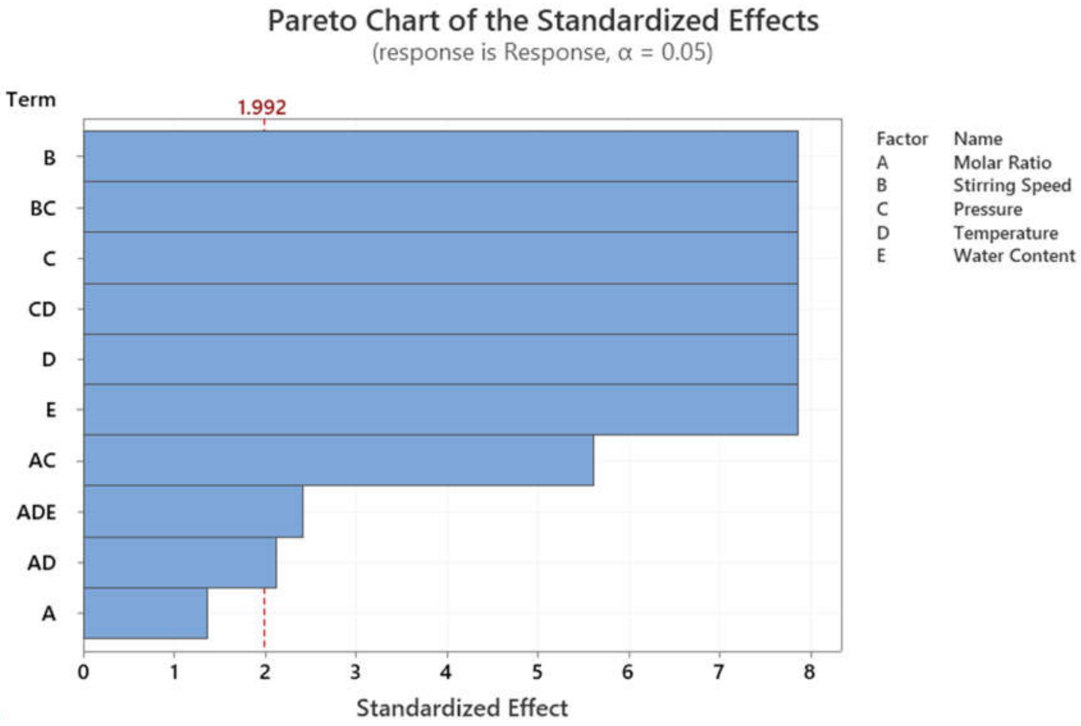

z

Figure S8. Pareto chart of the standardized effects after removing the insignificant terms

K. Refined model equation is presented as per Equation S9 below

Regression Equation

```
Response^0.279157 = 0.87842 - 0.001098 Molar Ratio_0.67 + 0.001098 Molar Ratio_0.75
                    - 0.20823 Stirring Speed_0 + 0.02383 Stirring Speed_50
                    + 0.09216 Stirring Speed_100 + 0.09224 Stirring Speed_250
                    - 0.29990 Pressure_1 - 0.10561 Pressure_2 + 0.02151 Pressure_3
                    + 0.08885 Pressure_4 + 0.13812 Pressure_5 + 0.15702 Pressure_6
                    + 0.03453 Temperature_25 + 0.00087 Temperature_35
                    - 0.03540 Temperature_45 - 0.01349 Water Content_0.0
                    + 0.01270 Water Content_2.5 + 0.00079 Water Content_5.0
                    + 0.01063 Molar Ratio*Pressure_0.67 1 + 0.00348 Molar Ratio*Pressure_0.67
                    2 - 0.00040 Molar Ratio*Pressure_0.67 3
                    - 0.00364 Molar Ratio*Pressure_0.67 4 - 0.00469 Molar Ratio*Pressure_0.67
                    5 - 0.00537 Molar Ratio*Pressure_0.67 6
                    - 0.01063 Molar Ratio*Pressure_0.75 1 - 0.00348 Molar Ratio*Pressure_0.75
                    2 + 0.00040 Molar Ratio*Pressure_0.75 3
                    + 0.00364 Molar Ratio*Pressure_0.75 4 + 0.00469 Molar Ratio*Pressure_0.75
                    5 + 0.00537 Molar Ratio*Pressure_0.75 6
                    - 0.00101 Molar Ratio*Temperature_0.67 25
                    - 0.00193 Molar Ratio*Temperature_0.67 35
                    + 0.00294 Molar Ratio*Temperature_0.67 45
                    + 0.00101 Molar Ratio*Temperature_0.75 25
                    + 0.00193 Molar Ratio*Temperature_0.75 35
                    - 0.00294 Molar Ratio*Temperature_0.75 45
                    - 0.09758 Stirring Speed*Pressure_0 1 - 0.08438 Stirring Speed*Pressure_0
                    2 + 0.01909 Stirring Speed*Pressure_0 3
                    + 0.04028 Stirring Speed*Pressure_0 4 + 0.06315 Stirring Speed*Pressure_0
                    5 + 0.05943 Stirring Speed*Pressure_0 6
                    + 0.03083 Stirring Speed*Pressure_50 1
                    + 0.03464 Stirring Speed*Pressure_50 2
                    - 0.00559 Stirring Speed*Pressure_50 3
                    - 0.01275 Stirring Speed*Pressure_50 4
                    - 0.02295 Stirring Speed*Pressure_50 5
                    - 0.02419 Stirring Speed*Pressure_50 6
                    + 0.03634 Stirring Speed*Pressure_100 1
                    + 0.02687 Stirring Speed*Pressure_100 2
                    - 0.00536 Stirring Speed*Pressure_100 3
                    - 0.01432 Stirring Speed*Pressure_100 4
                    - 0.02160 Stirring Speed*Pressure_100 5
                    - 0.02193 Stirring Speed*Pressure_100 6
                    + 0.03041 Stirring Speed*Pressure_250 1
                    + 0.02287 Stirring Speed*Pressure_250 2
                    - 0.00815 Stirring Speed*Pressure_250 3
                    - 0.01322 Stirring Speed*Pressure_250 4
                    - 0.01860 Stirring Speed*Pressure_250 5
                    - 0.01331 Stirring Speed*Pressure_250 6 + 0.04840 Pressure*Temperature_1
                    25 - 0.01460 Pressure*Temperature_1 35 - 0.03379 Pressure*Temperature_1
                    45 + 0.00369 Pressure*Temperature_2 25 + 0.01577 Pressure*Temperature_2
                    35 - 0.01947 Pressure*Temperature_2 45 - 0.00614 Pressure*Temperature_3
                    25 + 0.01045 Pressure*Temperature_3 35 - 0.00431 Pressure*Temperature_3
                    45 - 0.00200 Pressure*Temperature_4 25 - 0.00260 Pressure*Temperature_4
                    35 + 0.00460 Pressure*Temperature_4 45 - 0.01748 Pressure*Temperature_5
                    25 - 0.00325 Pressure*Temperature_5 35 + 0.02073 Pressure*Temperature_5
                    45 - 0.02647 Pressure*Temperature_6 25 - 0.00577 Pressure*Temperature_6
                    35 + 0.03224 Pressure*Temperature_6 45
                    - 0.00201 Molar Ratio*Temperature*Water Content_0.67 25 0.0
                    + 0.00143 Molar Ratio*Temperature*Water Content_0.67 25 2.5
                    + 0.00058 Molar Ratio*Temperature*Water Content_0.67 25 5.0
                    - 0.00241 Molar Ratio*Temperature*Water Content_0.67 35 0.0
                    + 0.00371 Molar Ratio*Temperature*Water Content_0.67 35 2.5
                    - 0.00130 Molar Ratio*Temperature*Water Content_0.67 35 5.0
                    + 0.00442 Molar Ratio*Temperature*Water Content_0.67 45 0.0
                    - 0.00514 Molar Ratio*Temperature*Water Content_0.67 45 2.5
                    + 0.00072 Molar Ratio*Temperature*Water Content_0.67 45 5.0
                    + 0.00201 Molar Ratio*Temperature*Water Content_0.75 25 0.0
                    - 0.00143 Molar Ratio*Temperature*Water Content_0.75 25 2.5
                    - 0.00058 Molar Ratio*Temperature*Water Content_0.75 25 5.0
                    + 0.00241 Molar Ratio*Temperature*Water Content_0.75 35 0.0
                    - 0.00371 Molar Ratio*Temperature*Water Content_0.75 35 2.5
                    + 0.00130 Molar Ratio*Temperature*Water Content_0.75 35 5.0
                    - 0.00442 Molar Ratio*Temperature*Water Content_0.75 45 0.0
                    + 0.00514 Molar Ratio*Temperature*Water Content_0.75 45 2.5
                    - 0.00072 Molar Ratio*Temperature*Water Content_0.75 45 5.0
```

Equation S9. Refined model equation

L. Factorial design array

Table S1. Factorial design array

Factor Information

Factor	Levels	Values
Molar Ratio	2	0.67, 0.75
Stirring Speed	4	0, 50, 100, 250
Pressure	6	1, 2, 3, 4, 5, 6
Temperature	3	25, 35, 45
Water Content	3	0.0, 2.5, 5.0

M. Interaction's plot

The second-order interactions of parameters have a total contribution in the model with 2.53 % However, in terms of significance, pressure with temperature interaction is the most significant interaction (P-value = 0.001 < 0.05) followed by stirring speed with pressure interactions and pressure with molar ratio interactions respectively among second-way interactions as per figure S9 below. The interactions among molar ratio, temperature and water content are the only significant third-order interaction. Whereas, the rest of the second-order and third-order interactions are insignificant (P-values > 0.05) along with the fourth-order interactions as per figure S9. In detail, for any DESs composition CO_2 solubility in ChCl: LvAc DESs increases with increasing pressure, stirring speed, water content and molar ratio respectively. However, CO_2 solubility declines with increasing temperature as per Figure below.

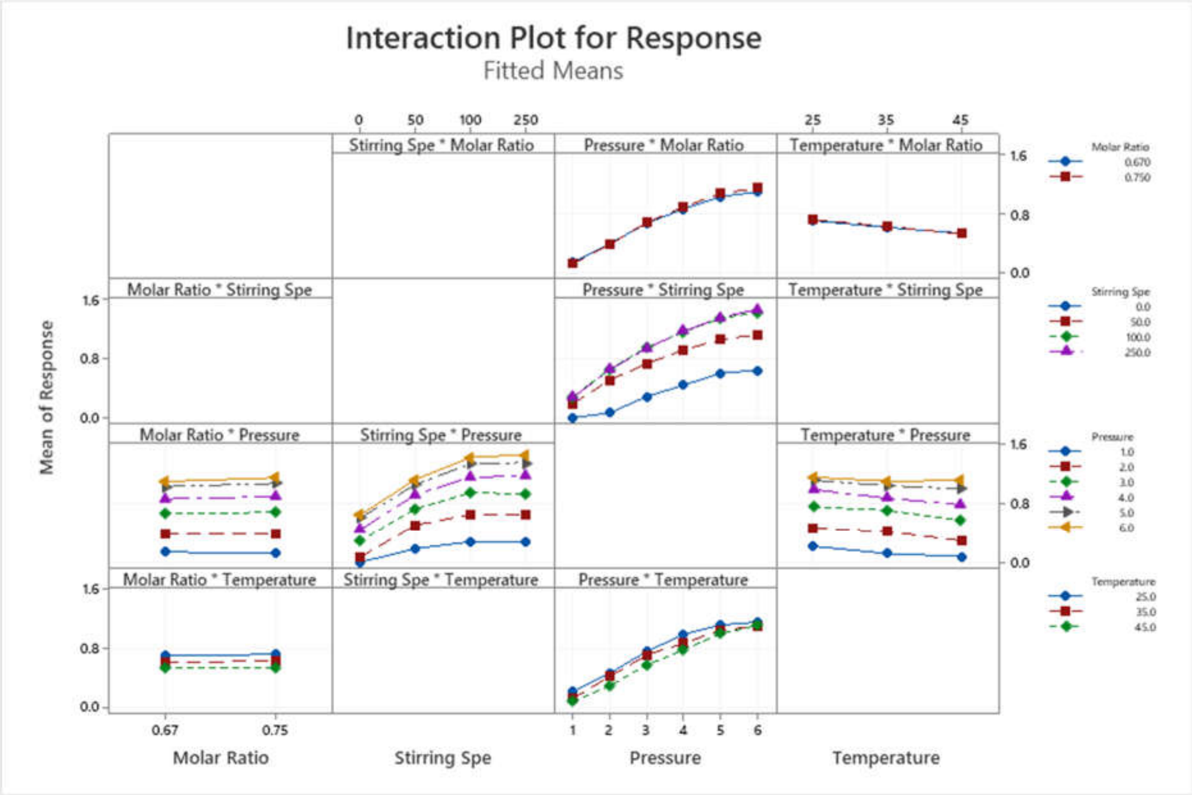

Figure S9. The second-order interactions plots

N. Optimisation of controlled parameters

The optimal operating parameters that give the highest CO_2 absorption by the DESs were prepared using Minitab optimizer as per Figure below. The optimal parameters of HBA: HBD molar ratio, pressure, and water contents found to be (1:3), 6 bar, 250 rpm and 2.5% respectively. Optimized values are consistent with the experimental results mentioned in the experimental section above and with the similar work reported in the literature for other DESs (29) & (38).

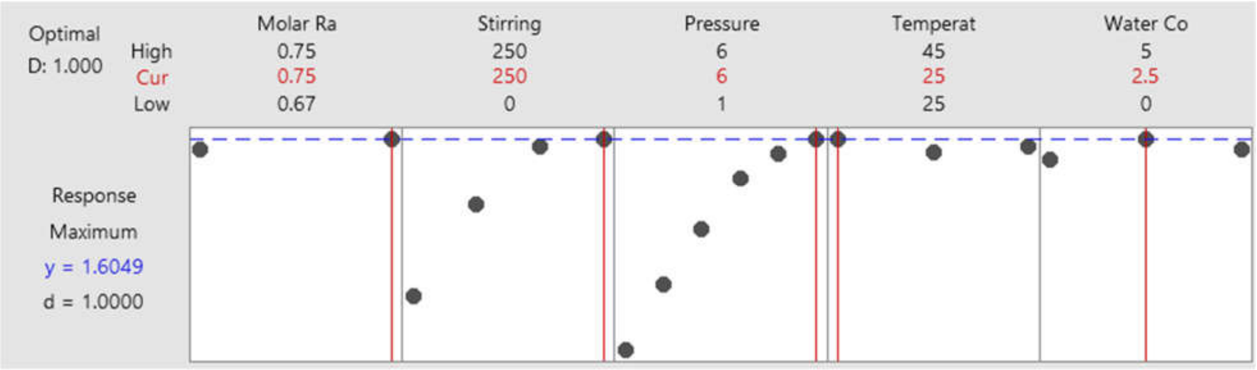

Figure S10. Optimization of controlled parameters

O. Prediction of CO_2 absorption by DESs

This model allows predicting the corresponding response of CO_2 absorption by the DESs at any given controlled parameters including high-pressure values these are unfeasible in the laboratory, or operating parameters from pilot/industrial plants.

P. Model applicability

CO_2 absorption data in ChCl: LvAc DESs were prepared isothermally at temperatures up to 318.15 K with different levels of other factors according to equation S9 in the supplementary information document. To apply this model, absorption data should be prepared within these ranges of temperature, pressure, stirring speed, molar ratio and water content.

Q. Residual's analysis

The residual plot for the data shows normal distribution of the residuals with equal variance and independent distribution as shown below in Figure . Which validate that the fitted model is highly accurate with $R^2 > 99\%$ as shown in section F the supplementary information document.

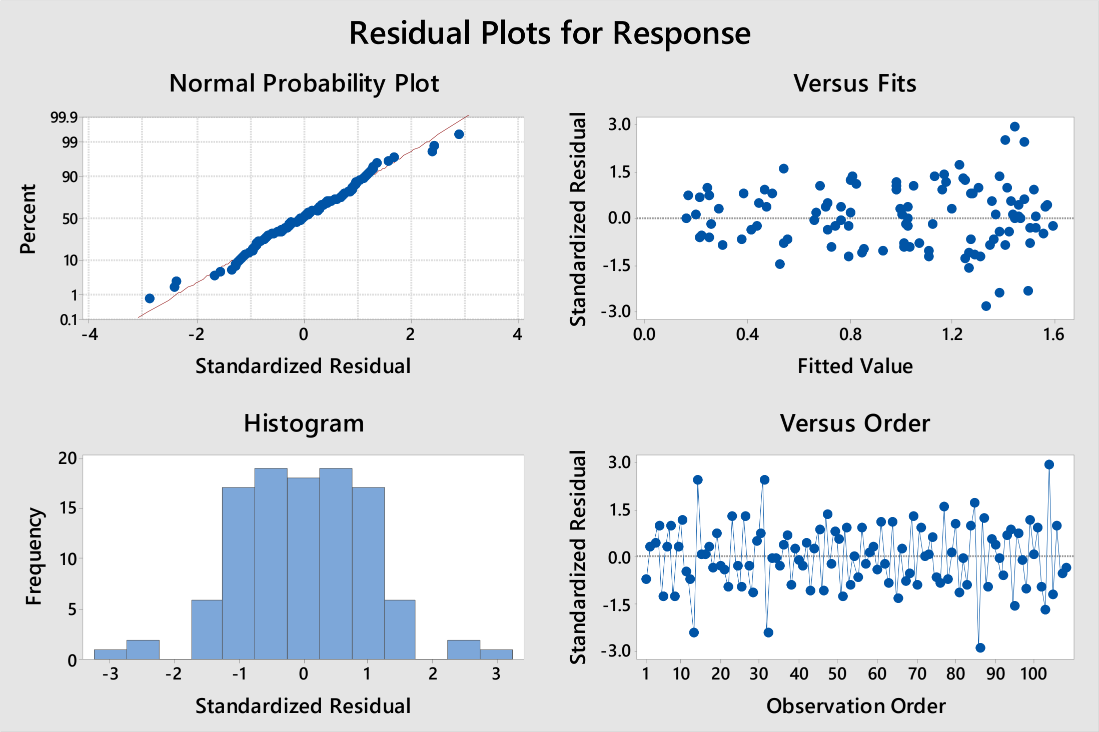

Figure S11. Residual plots for response

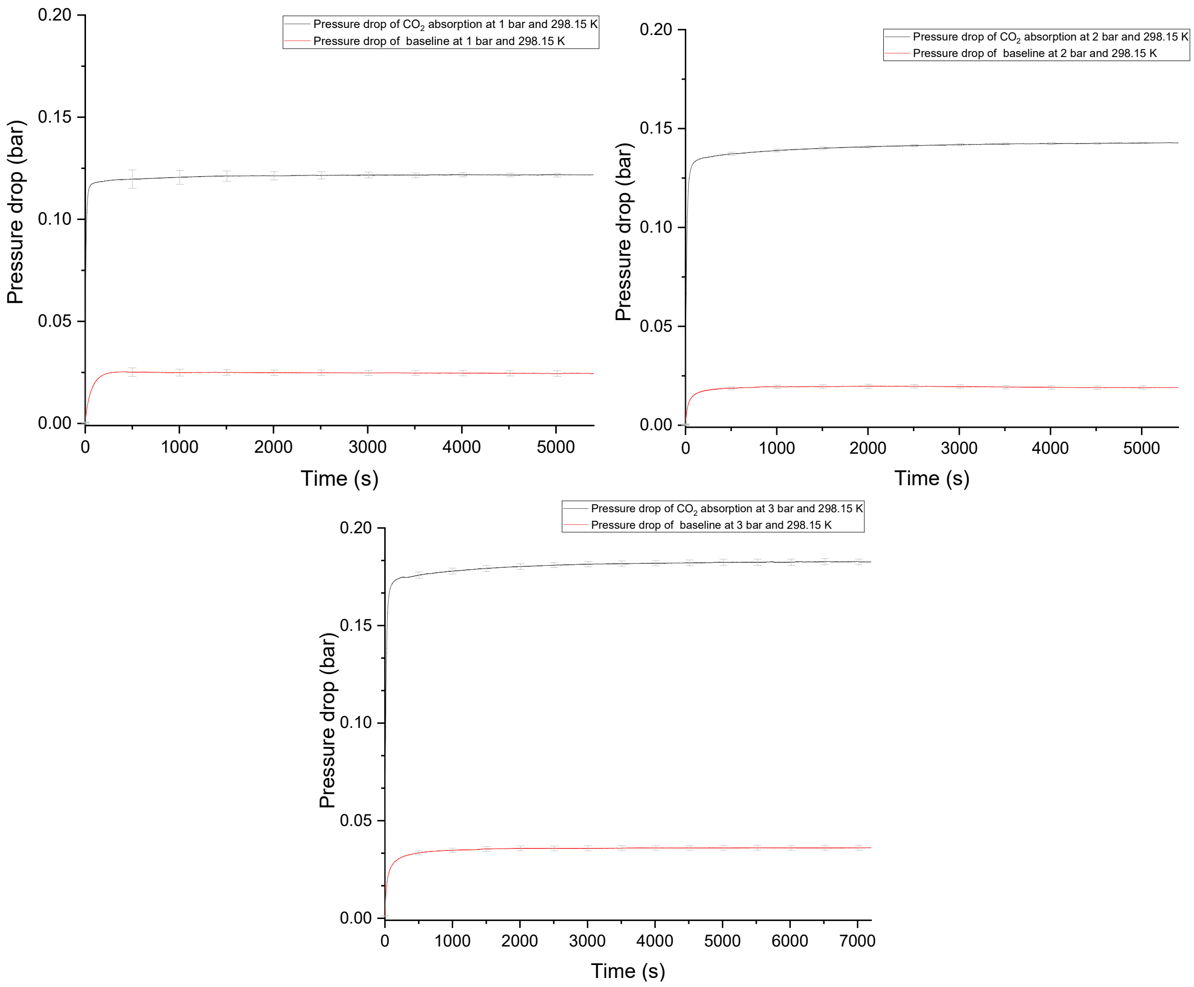

Figure S12a. Pressure drop values of the absolute pressure drop in the rig due to CO_2 absorption by chcl:lvac of (1:2:0) molar ratio at 25 °C and 250 rpm of the first cycle Vs. the pressure drop of the baselines

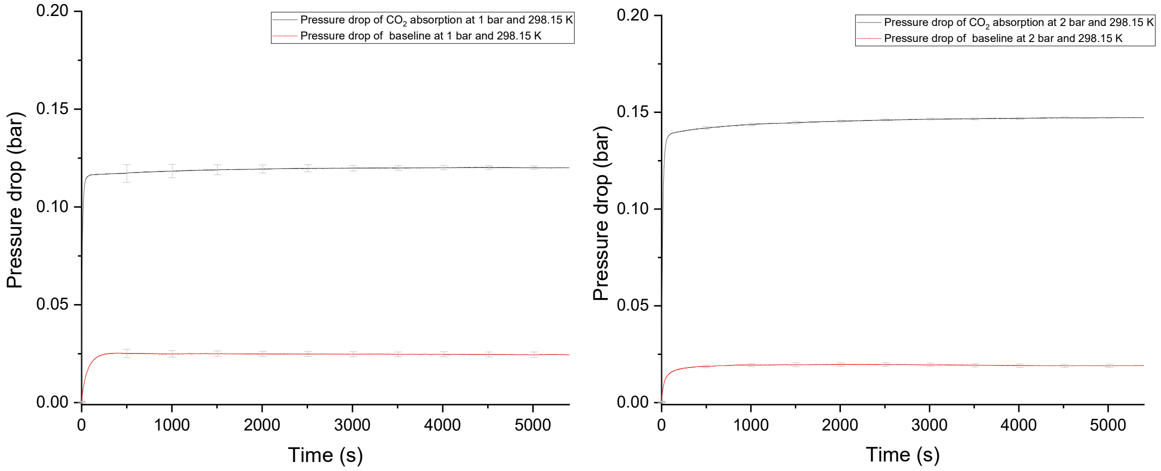

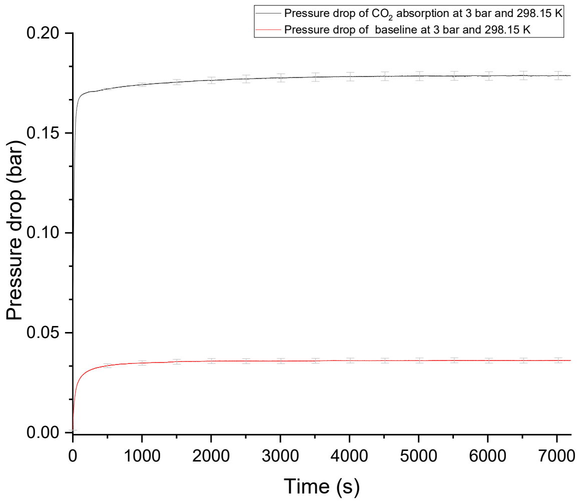

Figure S12b. Pressure drop values of the absolute pressure drop in the rig due to CO_2 absorption by chcl:lvac of (1:2:0) molar ratio at 25 °C and 250 rpm of the second cycle Vs. the pressure drop of the baselines

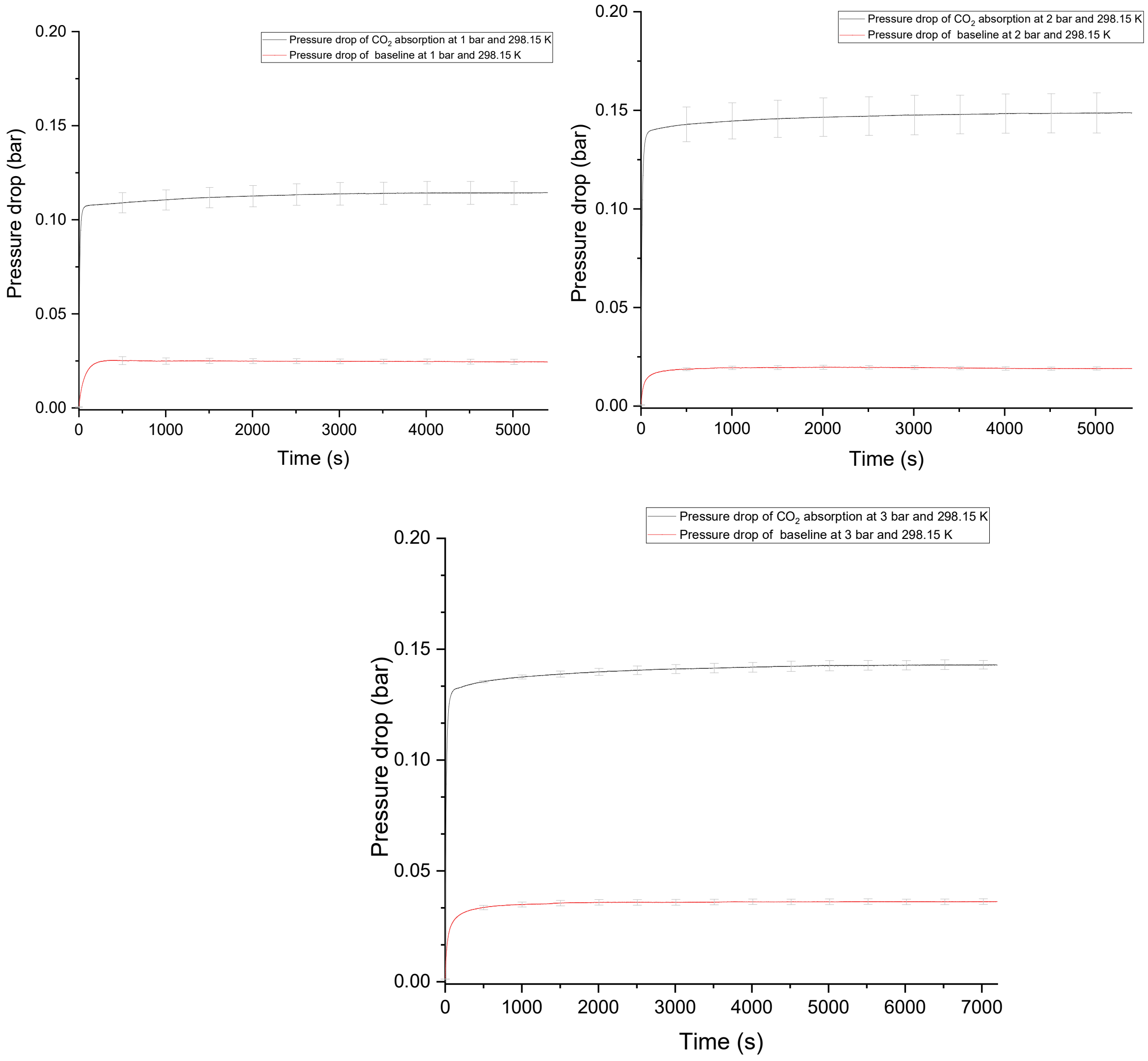

Figure S12c. Pressure drop values of the absolute pressure drop in the rig due to CO_2 absorption by chcl:lvac of (1:2:0) molar ratio at 25 °C and 250 rpm of the third cycle Vs. the pressure drop of the baselines

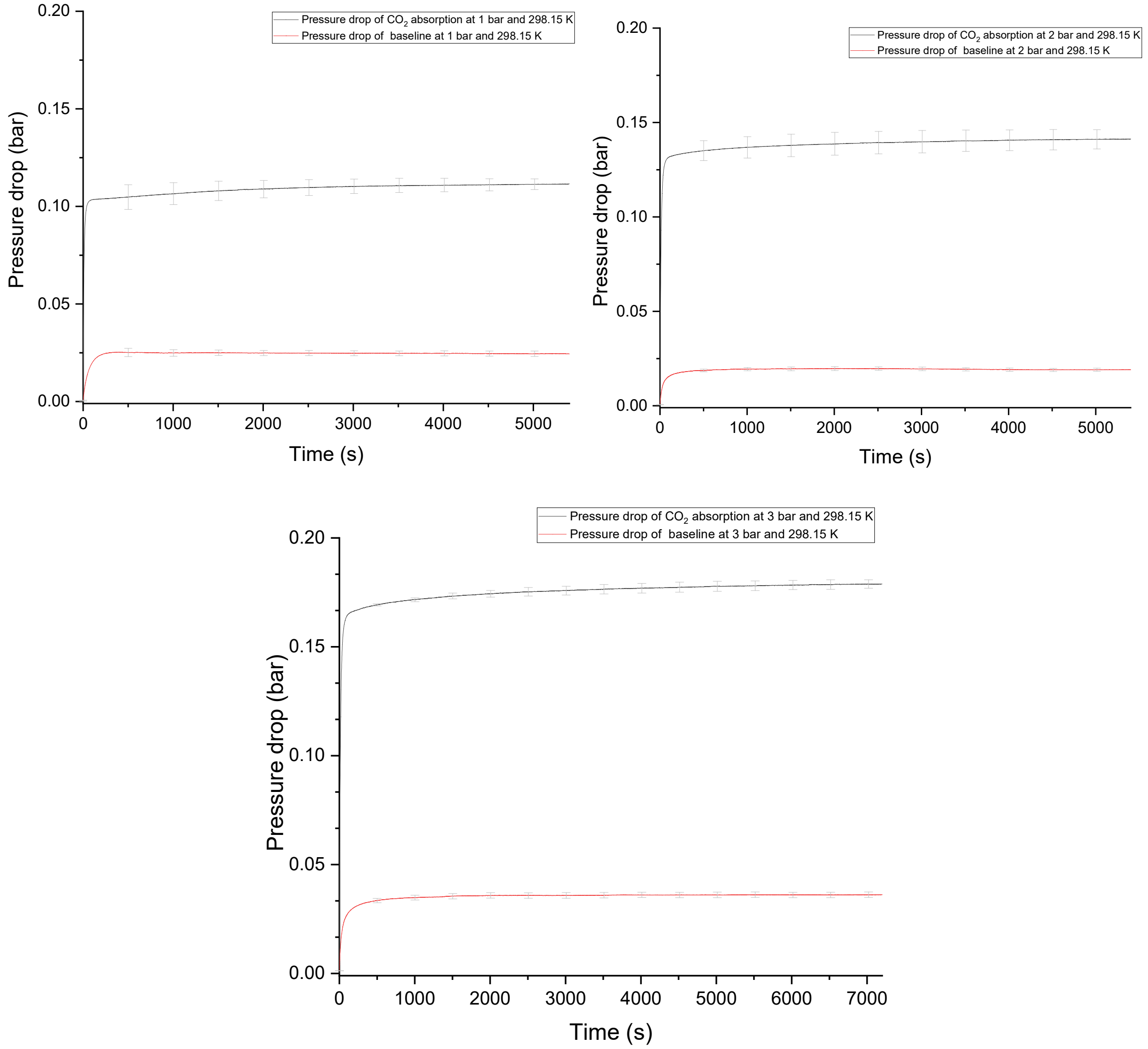

Figure S12d. Pressure drop values of the absolute pressure drop in the rig due to CO_2 absorption by chcl:lvac of (1:2:0) molar ratio at 25 °C and 250 rpm of the fourth cycle Vs. the pressure drop of the baselines

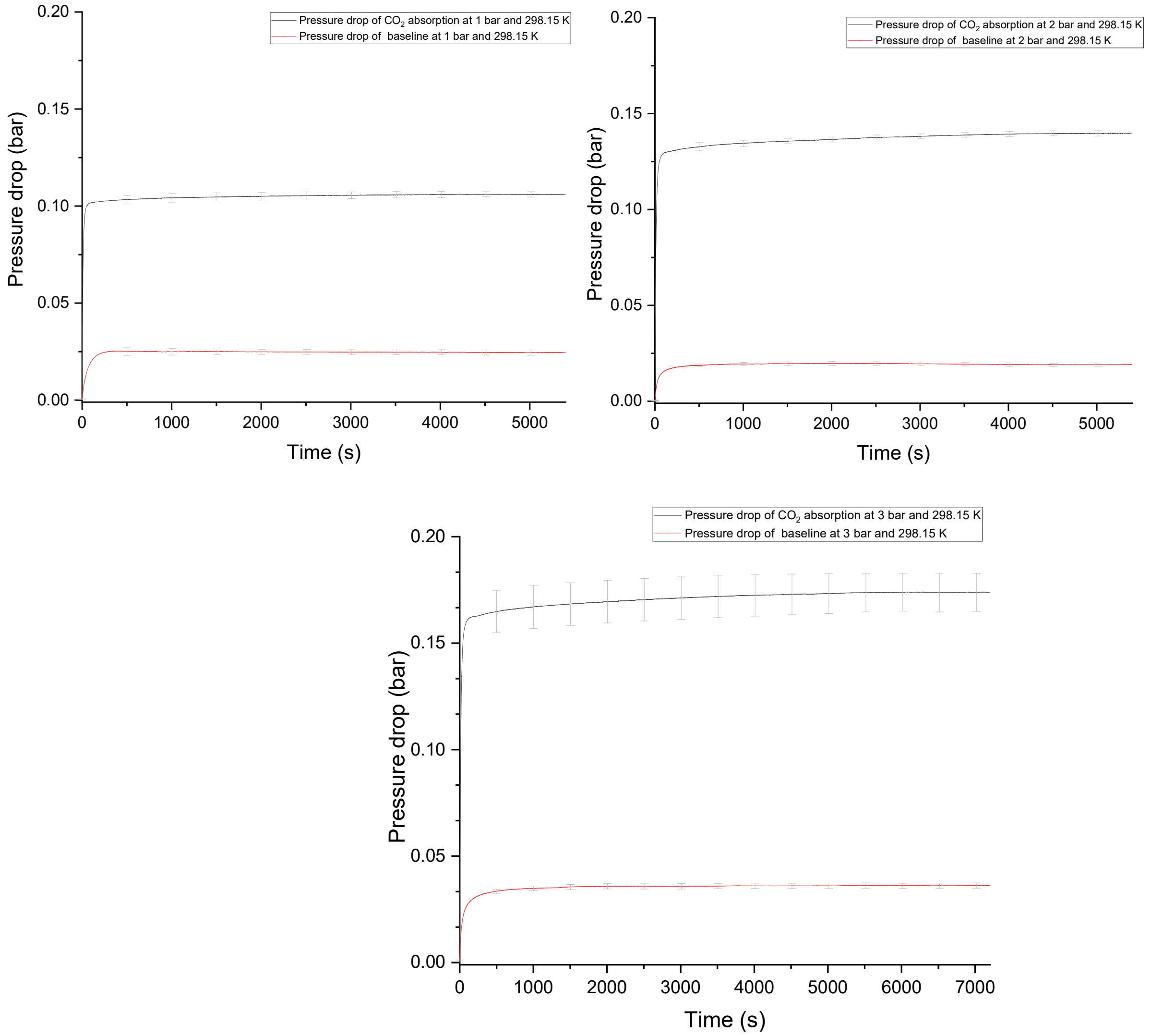

Figure S12e. Pressure drop values of the absolute pressure drop in the rig due to CO_2 absorption by chcl:lvac of (1:2:0) molar ratio at 25 °C and 250 rpm of the fifth cycle Vs. the pressure drop of the baselines

The VLE rig reaches equilibrium within 2 hours as shown in figure S13 below. The absolute pressure drop due to the CO_2/N_2 absorption only is the difference between the two isotherms pressure drop curves in figure S13 below.

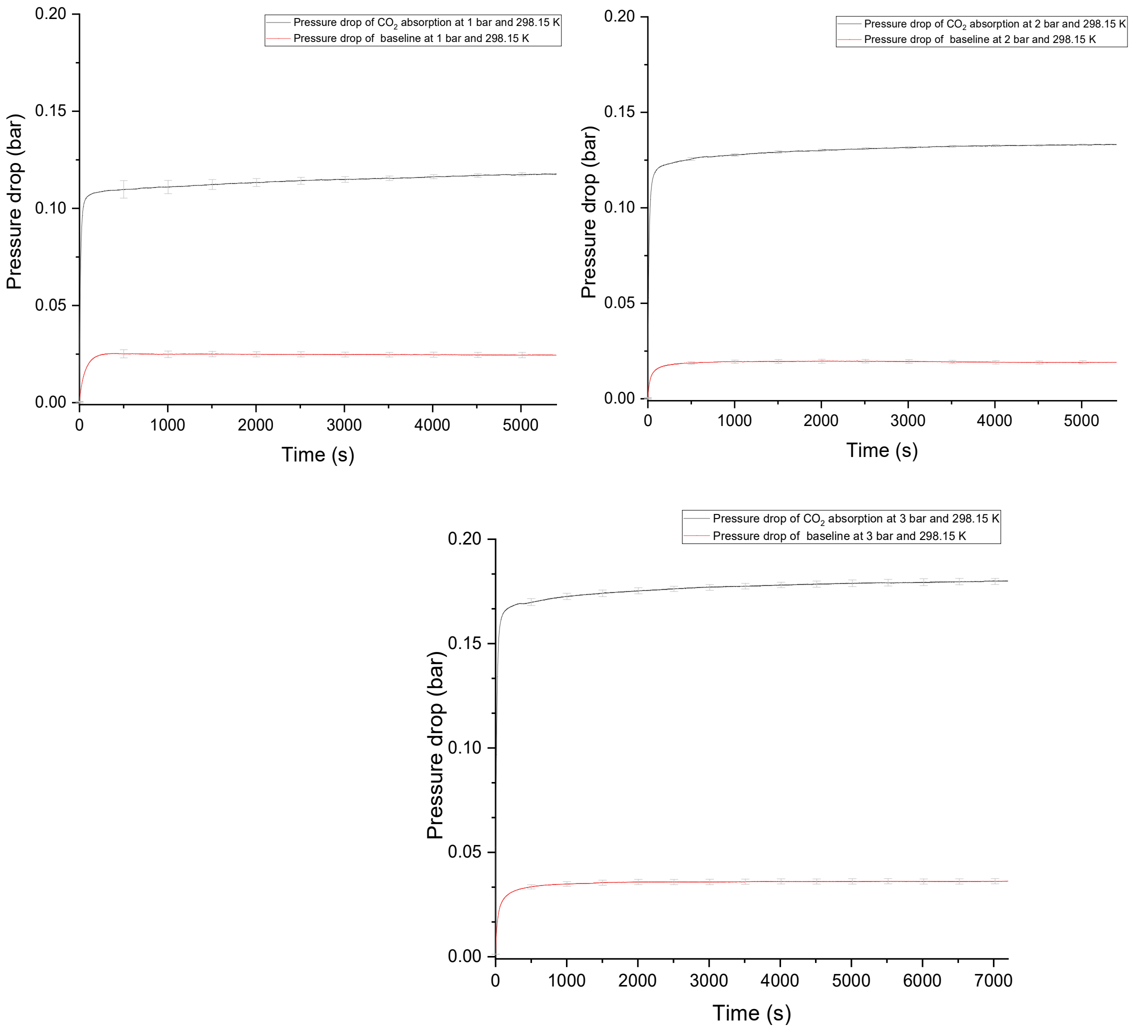

Figure S13a. CO_2 absorption raw data for tests curried out with a chcl:lvac DES with molar ratio (1:3:2.5) and a gas mixture with 100% CO_2 and 0% N_2, tests curried out at 25 °C and stirring speed of 250 rpm

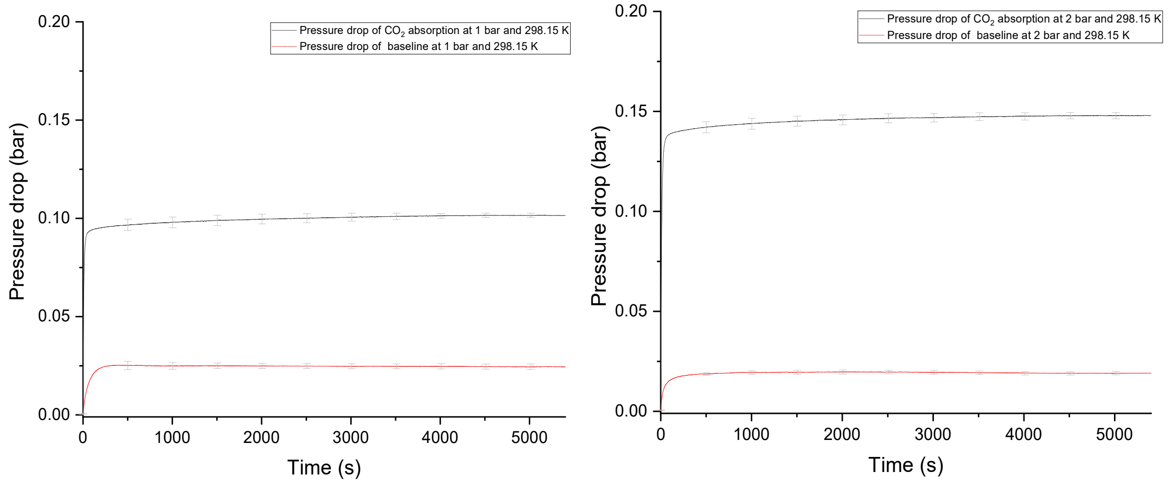

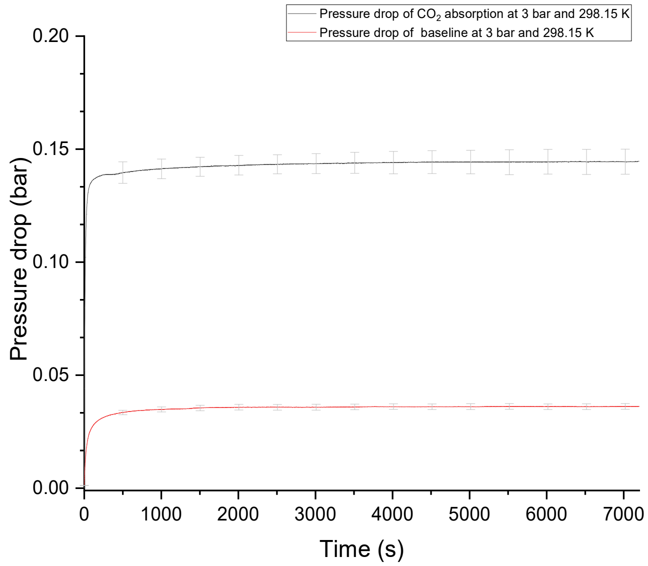

Figure S13b. CO_2 absorption raw data for tests curried out with a chcl:lvac DES with molar ratio (1:3:2.5) and a gas mixture with 50% CO_2 and 50% N_2, tests curried out at 25 °C and stirring speed of 250

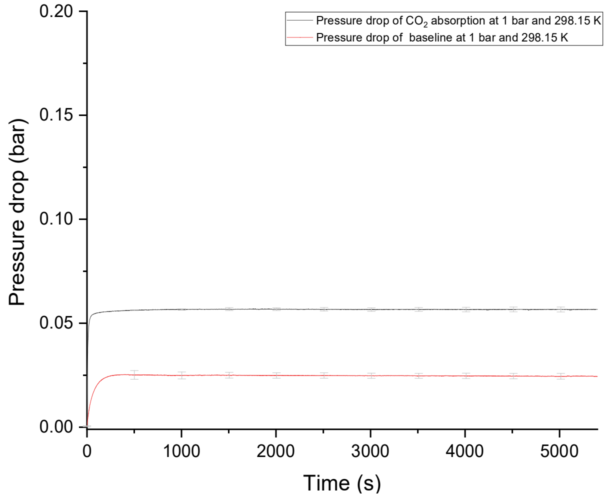

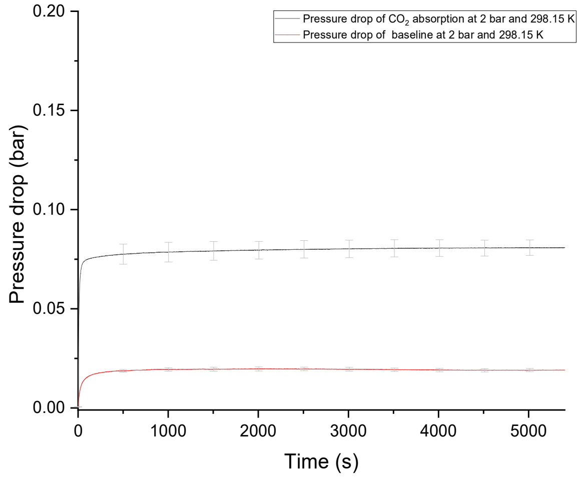

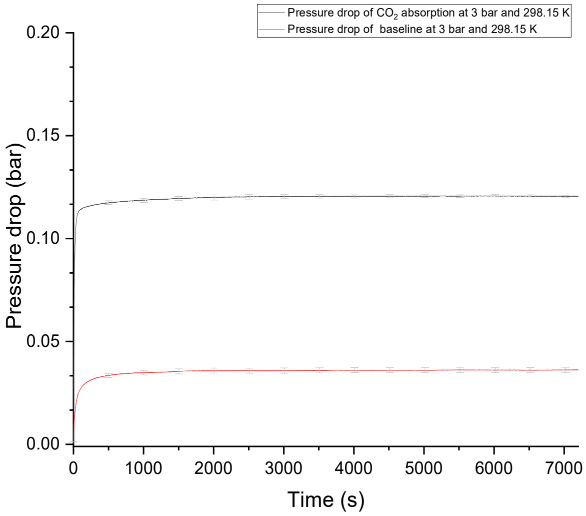

Figure S13c. CO_2 absorption raw data for tests curried out with chcl:lvac DES with molar ratio (1:3:2.5) and a gas mixture with 15% CO_2 and 85% N_2, tests curried out at 25 °C and stirring speed of 250 rpm

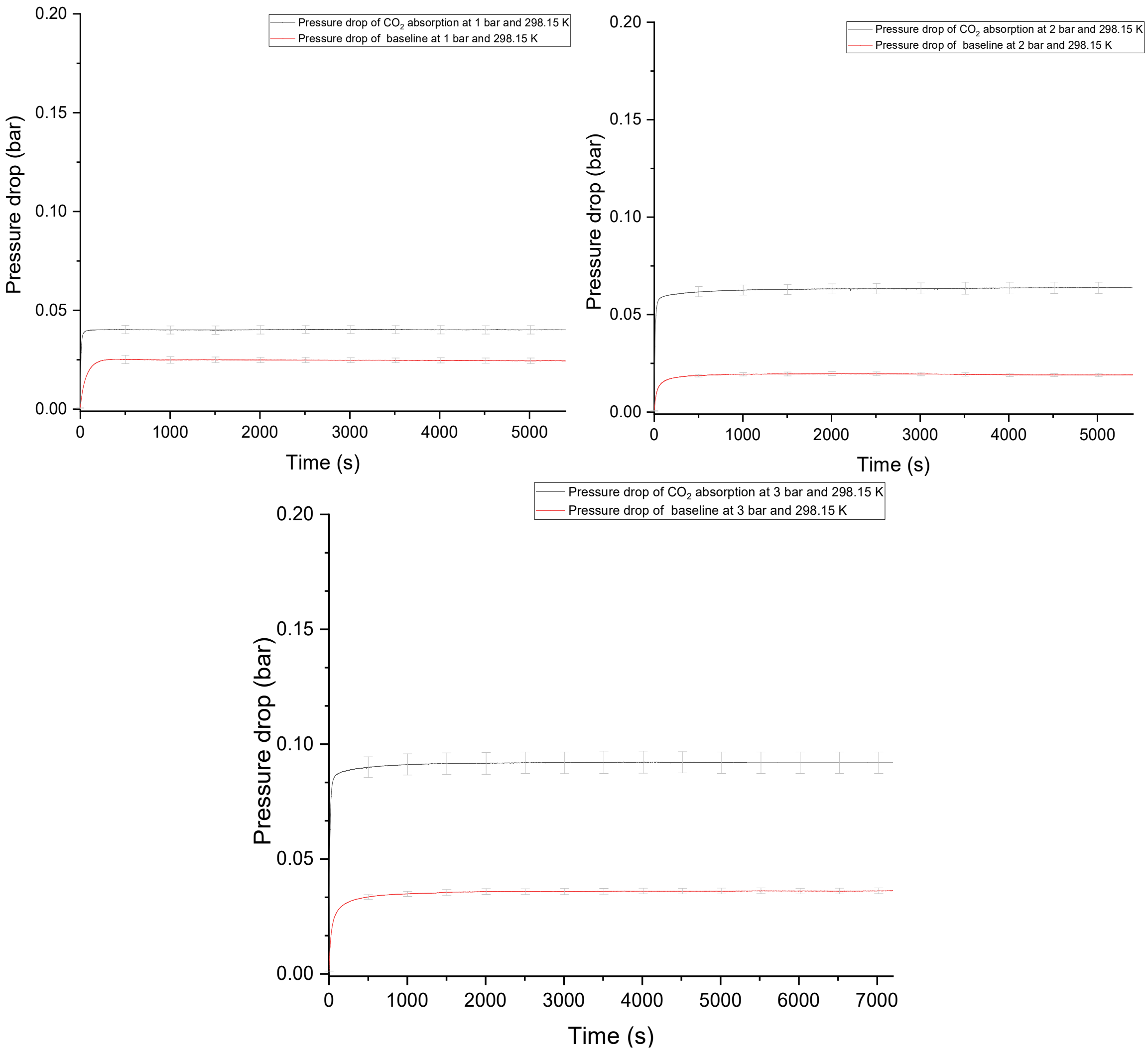

Figure S13d. CO_2 absorption raw data for tests curried out with a chcl:lvac DES with molar ratio (1:3:2.5) and a gas mixture with 0% CO_2 and 100% N_2, tests curried out at 25 °C and stirring speed of 250 rpm.

References

1. Lee JI, Otto FD, Mather AE. Equilibrium Between Carbon Dioxide and Aqueous Monoethanolamine Solutions. J Appl Chem Biotechnol. 1976;26(10):541–9.

2. Jou F, Mather AE, Otto FD. The Solubility of CO2 in a 30 Mass Percent MEA Solution. Can J CHEM ENG VOL73 1995. 1995;73.

3. Leron RB, Li MH. Solubility of carbon dioxide in a choline chloride-ethylene glycol based deep eutectic solvent. Thermochim Acta [Internet]. 2013;551:14–9. Available from: http://dx.doi.org/10.1016/j.tca.2012.09.041

4. Hasib-ur-Rahman M, Siaj M, Larachi F. Ionic liquids for CO2capture-Development and progress. Chem Eng Process Process Intensif. 2010;49(4):313–22.

5. Li G, Deng D, Chen Y, Shan H, Ai N. Solubilities and thermodynamic properties of CO2 in choline-chloride based deep eutectic solvents. J Chem Thermodyn [Internet]. 2014;75:58–62. Available from: http://linkinghub.elsevier.com/retrieve/pii/S0021961414001189

6. (CDIAC) CDIAC. Calculation of the fugacity of carbon dioxide in the pure gas or in air. Carbon Dioxide Inf Anal Cent [Internet]. 1997;(3):1–5. Available from: https://cdiac.ess-dive.lbl.gov/#

7. Wu SH, Caparanga AR, Leron RB, Li MH. Vapor pressure of aqueous choline chloride-based deep eutectic solvents (ethaline, glyceline, maline and reline) at 30-70 °c. Thermochim Acta [Internet]. 2012;544:1–5. Available from: http://dx.doi.org/10.1016/j.tca.2012.05.031
